# Stochastic-SplitGAS: A Quantum Monte Carlo Multi-Reference Perturbation Theory Based on the Imaginary-Time Evolution of Effective Hamiltonians

**DOI:** 10.1021/acs.jctc.5c01270

**Published:** 2025-12-03

**Authors:** Luca Bonfirraro, Oskar Weser, Maru Song, Giovanni Li Manni

**Affiliations:** † 28326 Max Planck Institute for Solid State Research, Heisenbergstr. 1, 70569 Stuttgart, Germany; ‡ Massachusetts Institute of Technology, 77 Massachusetts Ave, Cambridge, Massachusetts 02139, United States

## Abstract

Accurately modeling the electronic structure of systems with many unpaired electrons remains a major challenge in quantum chemistry. Qualitatively correct electronic structures generally require large active space multireference wave functions, while dynamic correlation effects beyond the active space are crucial for quantitatively accurate descriptions of magnetic, catalytic and optical properties of such systems. Here, we present an uncontracted multireference perturbation theory based on the FCIQMC imaginary-time evolution of effective Hamiltonians, built upon the generalized active space concept and Löwdin’s partitioning technique. The configurational interaction space is split into a reference space, consisting of the most important configurations, and a perturber space, containing the more numerous configurations responsible for dynamic correlation effects. The generalized active space algorithm allows the flexible partitioning of the configurational space. Löwdin’s partitioning technique is then used to construct an effective Hamiltonian which is stochastically solved. This strategy allows us to apply perturbative corrections on large active space reference wave functions, without requiring high-order reduced density matrices, which have been found the bottleneck in other perturbation theory strategies. The capabilities of the resulting method, called Stochastic-SplitGAS, are demonstrated on the triplet-quintet spin gap of an Fe­(II)-porphyrin model system and the spin ladder of a [Fe­(III)_2_S_2_]^2–^ complex.

## Graphical abstract

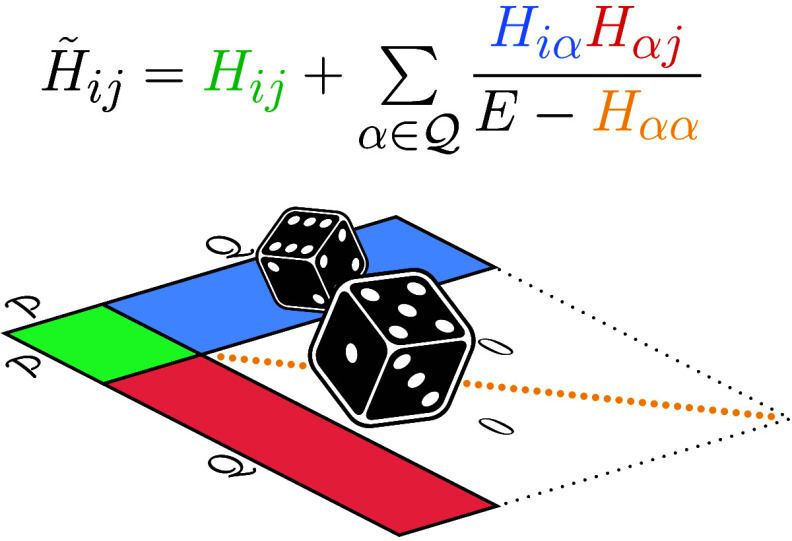

## 1 Introduction

The complete active space self-consistent field method, CASSCF, in its original formulation,^1–6^ is a well established multireference strategy to obtain qualitatively accurate electronic wave functions for strongly correlated systems. However, due to its computational cost, its applicability is limited to small active spaces, typically containing less than 20 electrons in 20 orbitals, CAS­(20,20).^7^ In the past 20 years, the scope of the active space strategy has been extended by the introduction of the density matrix renormalization group (DMRG) approach,^8–23^ full-CI quantum Monte Carlo (FCIQMC),^24–27^ Stochastic-CASSCF,^28,29^ the generalized active space (GAS) method in its deterministic^30^ and stochastic^31^ formulations, the occupation-restricted-multiple-active-space (ORMAS),^32^ the localized active spaces (LAS),^33–38^ and selected configuration interaction (Selected-CI) approaches.^39–54^ Relevant is also the development of active space decomposition DMRG (ASD-DMRG),^55^ neural network-based Quantum Monte Carlo (NNQMC),^56^ and nonorthogonal approaches.^57–63^ Exemplary is the nonorthogonal configuration-interaction (NOCI) method recently developed in the GronOR codebase,^64–67^ efficient in the investigation of molecular systems with minimal overlap among the magnetic orbitals (or delocalized fragments orbitals). These strategies have enabled the quantum chemistry community to efficiently cover substantially larger active spaces.^19,23,31,38,54,68–76^

Adding correlation effects beyond the active space (dynamic correlation) is a crucial step to obtain quantitatively accurate electronic structure energetics. Perturbation-theory approaches, such as CASPT2,^77,78^ RASPT2,^79,80^ GASPT2 ^81^, ORMAS-PT,^209^ and NEVPT2 ^82–87^ are widely used to treat this form of electron correlation. However, in their *contracted* form, these methodologies rely on three- and four-body reduced density matrices (RDMs), which are computationally demanding and limit their applicability to small active spaces. More advanced methods such as FR-NEVPT2 can require up to fifth-order RDMs, although there have been advances to reduce this bottleneck.^88–90^ Recent developments such as the adiabatic connection AC0 and its variants,^91–94^ and NEVPT within singles (NEVPTS) by Pernal and co-workers^95^ reduce this cost as they circumvent the need for higher-order RDMs. Work has been reported to couple perturbation theory to preceding large active space zeroth-order wave functions, including DMRG-CASPT2,^96–101^ and stochastic second-order perturbation theory.^102–105^ In the context of Stochastic-CASPT2, zeroth-order wave functions corresponding to active spaces as large as CAS­(26,27) have been reported, with the three- and four-body density matrices, including contractions with the Fock matrix, computed from imaginary-time-averaged wave functions.^105^ Similar sizes of active spaces have been reported in the context of DMRG-CASPT2.^99^

Multiconfiguration pair-density functional theory, MC-PDFT,^106,107^ has been proposed as an inexpensive alternative to PT2 to treat dynamic correlation effects. The method depends solely on the one- and two-body RDMs, and it is thus compatible with large active space reference wave functions, as the more expensive evaluation of high-order density matrices is avoided. MC-PDFT has been successfully coupled to large active space methods, including DMRG^108^ and v2RDM-CASSCF.^109^ Recently, we have applied the combined Stochastic-CAS (and DMRG) plus MC-PDFT approach to the large CAS­(56,56) active space of a strongly correlated Co­(II)_3_Er­(III)­OR_4_ complex, predicting a ferromagnetic ordering of the low-energy spin states for the system.^70^

*Uncontracted* perturbation theory also avoids high-order RDMs, albeit at the cost of explicitly evaluating electron excitations relative to all electronic configurations of the zeroth-order reference wave function. Their computational cost scales linearly with the number of configurations in the zeroth-order wave function, and in the case of large active space wave functions the perturbative correction becomes computationally prohibitive. Previous work towards a stochastic implementation of uncontracted perturbation theory has been made in the form of LCCQMC.^210^

In the present work, a stochastic uncontracted multireference perturbation theory strategy is presented that combines Löwdin’s partitioning technique^110^—also referred to as *downfolding*—truncated to the second order with the Stochastic-GAS algorithm.^31^ We demonstrate that the stochastic strategy largely circumvents the unfavorable scaling of uncontracted perturbation theory. The new paradigm allows us to efficiently treat the effect of the perturber space on the zeroth-order reference wave function without requiring high-order density matrices, and by largely circumventing the limits reported for contracted perturbation theory.^98,104,105^ The nonstochastic version of the method has been previously implemented in the context of the GAS method and is referred to as SplitGAS;^111^ thus, we refer to the novel stochastic version as *Stochastic-SplitGAS*. The theoretical framework of the method is outlined in Section 2, and its capabilities are demonstrated in Section 3 where two test cases are presented: the spin ladder of a [Fe­(III)_2_S_2_]^2–^ model system, and the triplet-quintet spin gap of an Fe­(II)-porphyrin complex, where a total of 96 electrons are correlated in 159 orbitals with a CAS­(32,34) reference space.

## 2 Theory

In this section, theoretical concepts that are propaedeutic to the Stochastic-SplitGAS implementation are outlined, namely (a) the FCIQMC algorithm, which is used to stochastically solve large active space wave functions, both with and without perturbative correction, (b) the GAS concept, which enables flexible partitioning of the configurational space into principal and perturber spaces, particularly in its stochastic formulation,^31^ and (c) Löwdin’s partitioning technique in its SplitGAS formulation,^111^ which leads to the working equation for the perturbative correction.

### 2.1 The FCIQMC Algorithm

A detailed explanation of the FCIQMC algorithm can be found in the literature,^24,25^ while only an outline of the main steps which are necessary to understand the key concepts of Stochastic-SplitGAS is given here.

Starting from the imaginary-time Schrödinger equation

1 $$\frac{\partial }{\partial \tau }\left|\Psi \left(\tau \right)\right\rangle =-Ĥ\left|\Psi \left(\tau \right)\right\rangle$$

which is formally integrated to

2 |Ψ(τ)⟩=exp(−τĤ)|Ψ̃(τ=0)⟩

and a trial wave function, |Ψ̃(τ = 0)⟩, with nonvanishing overlap with the ground state wave function, the imaginary-time dynamic reveals, at the long-time limit, the ground state wave function, |Ψ_0_⟩ following an exponential decay

3 $$\left|\Psi_{0}\right\rangle =\lim_{\tau \to \infty }\left|\mathrm{\Psi ̃}\left(\tau \right)\right\rangle$$

Linearization of the propagator, e^–τ*Ĥ*^, and discrete representation of the wave function, |Ψ̃(τ)⟩ = ∑_*i*_ *c*_*i*_(τ)|ϕ_*i*_⟩ leads to the following expression

4 $$\Delta c_{j}=-\Delta \tau \left(\underset{\mathrm{Spawn}}{\underset{︸}{\sum_{i,i\neq j}K_{\mathrm{ij}}c_{i}}}+\underset{\mathrm{Death}}{\underset{︸}{\left(K_{\mathrm{jj}}-S_{\tau }\right)c_{j}}}\right)$$

where the energy of the reference configuration, *E*_ref_, has been removed from the diagonal terms of the Hamiltonian operator, (**K** = **H** – **I** *E*_ref_). Equation 4 describes the imaginary-time evolution of the variational parameters, Δ*c*_*j*_ = *c*_*j*_(τ + Δ*τ*) – *c*_*j*_(τ), of the wave function |Ψ­(τ)⟩. Equation 4 can be solved exactly only for active spaces correlating up to about 18 electrons in 18 orbitals. For larger active spaces it rapidly becomes computationally infeasible, due to the prohibitive costs of computing the extremely large number of *K*_*ij*_ elements, process them and store the *c*_*j*_(τ) coefficients in memory. In FCIQMC, the (unnormalized) wave function is represented by discretization of the *c*_*i*_ coefficients using *walkers* |Ψ­(τ)⟩ = ∑_*i*_ *n*_*i*_(τ)|ϕ_*i*_⟩, where *n*_*i*_(τ) is the number of walkers on configuration |ϕ_*i*_⟩ at the imaginary time τ. Only information on the many-body expansion that is populated by walkers at each imaginary-time step is stored, and the *K*_*ij*_ matrix elements are sampled stochastically, to avoid evaluation of all Hamiltonian matrix elements. The sparse representation through walkers enables the storage and imaginary-time evolution of large but sparse wave functions. The discrete representation of the wave function through a finite user-defined total number of walkers, *N*_walkers_, introduces a new parameter to control the convergence of the many-body wave function to the exact solution. In eq 4, the shift, *S*_τ_, is introduced to preserve the *L*_1_-norm of the wave function, i.e., it is used as a walker population control parameter. At stationarity, the shift *S*_τ_ is equal to the eigenvalue of the wave function, i.e., if the Hartree–Fock reference and the diagonally shifted Hamiltonian (**K** = **H** – **I** *E*_ref_) are used, then it equals the correlation energy.

At each imaginary-time step, Δ*τ*, the walker population is evolved in four stages: (i) excitation generation, (ii) spawning, (iii) death/cloning, and (iv) annihilation.

The *excitation generation* step lies at the heart of an efficient FCIQMC algorithm. Herein, new target configurations, |ϕ_*j*_⟩, are suggested for a given configuration, |ϕ_*i*_⟩, with a certain generation probability, *p*_gen_(*i, j*). In the simplest case, the selection is guided by a uniform generation probability, i.e., for a given parent configuration, |ϕ_*i*_⟩, any target configuration, |ϕ_*j*_⟩, is equally probable, as long as it is connected by a valid excitation.

The excitation generation process significantly impacts the efficiency of the FCIQMC algorithm. Ideally, *p*_gen_ should be chosen proportional to |*K*_*ij*_| such that the acceptance rate (*vide infra*) is nearly constant. An acceptance rate that is too low leads to a less efficient algorithm and increased autocorrelation. Conversely, a rate that is too high results in blooming events, where many walkers are spawned simultaneously, raising the standard deviation and destabilizing the dynamics. More efficient *weighted* excitation generators that suggest excitations with large *K*_*ij*_ more often have been developed.^25,41,112,113^ One example is the Pre-Computed Heat Bath (PCHB) excitation generator.^41^ Within PCHB, generation probabilities are assigned that are approximately proportional to the magnitude of the Hamiltonian matrix element connecting two configurations, |⟨ϕ_*i*_|*Ĥ*|ϕ_*j*_⟩|; therefore, strongly connected configurations are sampled more often than weakly connected ones.

For two Slater determinants (SDs), ϕ_*i*_ and ϕ_*j*_, connected by a double excitation |ϕ_*j*_⟩ = *Â* ^†^ *B̂* ^†^ *ÎĴ* |ϕ_i_⟩, the magnitude of the matrix element ⟨ϕ_*i*_|*Ĥ*|ϕ_*j*_⟩ does not depend on the involved determinants

5 $$\left|\left\langle \phi_{i}|Ĥ|\phi_{j}\right\rangle \right|=\left|g_{\mathrm{AIBJ}}-g_{\mathrm{AJBI}}\right|$$

instead, it only depends on the particle, *IJ*, and hole, *AB*, indices. Consequently, a determinant-independent weight, *W*_*IJ*_^*AB*^, is assigned to each excitation. In principle, these weights enable the calculation of the optimal excitation probabilities. However, exact probabilities derived from these weights are determinant-dependent.^114^ In PCHB a crucial approximation is made that eliminates the determinant-dependence of the excitation probabilities. This approximation enables the evaluation of all probabilities once at the beginning of the simulation (precomputed) which can then be sampled very efficiently in O(1) time using alias tables.^115,116^

For SDs connected by single excitations, the matrix element explicitly depends on the involved determinants, and single excitations have generally been sampled uniformly. When localized orbitals are used, single excitations gain importance, and their uniform sampling leads to suboptimal excitation generation. Recently, we have proposed a scheme that exploits locality and allows an approximated, but robust, definition of determinant-independent weights *S*_*I*_^*A*^

6 $$S_{I}^{A}=\{\begin{array}{ll} \left|h_{\mathrm{AI}}\right|+\sum_{R}\left|g_{\mathrm{AIRR}}-g_{\mathrm{ARRI}}\right| & I\neq A\\ 0 & \mathrm{else} \end{array}$$

Details can be found in the literature.^117^

In the spin-adapted basis within the Graphical Unitary Group Approach (GUGA),^118–120^ Hamiltonian matrix elements are evaluated as

7 $$\left\langle m\right|Ĥ\left|m^{\prime }\right\rangle =\sum_{\mathrm{ij}}h_{\mathrm{ij}}\left\langle m\right|{Ê}_{\mathrm{ij}}\left|m^{\prime }\right\rangle +\frac{1}{2}\sum_{\mathrm{ij},\mathrm{kl}}g_{\mathrm{ij},\mathrm{kl}}\left\langle m\right|{ê}_{\mathrm{ij},\mathrm{kl}}\left|m^{\prime }\right\rangle$$

where *m* and *m*′ are spin-adapted configuration state functions (CSFs), *Ê*_*ij*_ and *ê*_*ij,kl*_ are one- and two-body spin-free excitation operators, and *h*_*ij*_ and *g*_*ij*,*kl*_ are the spatial one- and two-electron integrals (in chemist’s notation). The matrix elements ⟨*m*|*Ê*_*ij*_|*m*′⟩ and ⟨*m*|*ê*_*ij,kl*_|*m*′⟩, which are independent of the integrals, are referred to as the GUGA coupling coefficients. Unlike in a SD basis, the coupling coefficients depend nontrivially on the coupled CSFs, making GUGA matrix element evaluation substantially more intricate. Accordingly, assigning CSF-independent weights is inherently nontrivial.

Thanks to the foundational work of Shavitt, Paldus, and others,^118,119,121,122^ analytical expressions for these coupling coefficients are available, enabling the derivation of approximate CSF-independent GUGA-PCHB weights for efficient stochastic spin-adapted CI-methods.^25,29,123,124^ The derivation of these approximate weights by some of us, to be discussed in a separate work,^125^ relies on two key observations. First, GUGA coupling coefficients are bounded, and for excitations involving small integrals, their contribution to the corresponding Hamiltonian matrix element becomes negligible. This effect is particularly pronounced in localized orbital bases, where the integrals *h*_*ij*_ and *g*_*ij*,*kl*_ decay exponentially with the distance between orbitals *i* – *j* and *k* – *l*. Second, different types of excitation operators exhibit qualitatively distinct behaviors. For example, the operator *ê*_*ij,ji*_ modifies only the spin coupling in the range between orbitals *i* and *j* (e.g., swapping *u* with *d* for orbitals between *i* and *j*), without altering orbital occupations, whereas a general excitation such as *ê*_*ij,kl*_, with all indices distinct, redistributes electrons among different spatial orbitals. These distinct excitation types lead to structurally different coupling coefficients and can thus be classified accordingly. These weights can be readily incorporated into Stochastic-GAS and Stochastic-SplitGAS, as shown in the present work.

Once |ϕ_*i*_⟩ and |ϕ_*j*_⟩ are drawn with a probability *p*_gen_, the *spawning* step takes place, where a number of new walkers are spawned from the parent configuration |ϕ_*i*_⟩ to the target configuration |ϕ_*j*_⟩. A spawn is accepted with an *acceptance probability* that is proportional to

8 $$p_{\mathrm{acc}}\left(i,j\right)=\Delta \tau \frac{\left|K_{\mathrm{ij}}\right|}{p_{\mathrm{gen}}\left(i,j\right)}$$

which depends on *p*_gen_. Equation 8 reveals how an excitation generator can improve both efficiency and stability of the simulation. If *p*_gen_(*i*, *j*) is proportional to |*K*_*ij*_|, the acceptance rate approaches a constant value, resulting in a more stable simulation that permits larger discrete time steps. The spawning step represents the stochastic version of the first summation in eq 4, leading to the Δ*c*_*j*_ update via the off-diagonal Hamiltonian coupling terms.

In the *death/cloning step*, all walkers on |ϕ_*j*_⟩ are killed or cloned proportionally to

9 $$p_{\mathrm{death}}\left(j\right)=\Delta \tau \left(K_{\mathrm{jj}}-S_{\tau }\right)$$

which is a stochastic manifestation of the second term in eq 4. In the *annihilation step*, parents and newly generated signed walkers are combined, where walkers of opposite sign on the same configuration eliminate each other.

In the *semistochastic approach*, stochastic propagation of the CI vector is performed alongside deterministic propagation of *n*_core_ selected configurations, typically those with high occupation. For all configurations in the *core space*, spawning and death steps are performed deterministically. Another noteworthy feature is that configurations within the core space are guaranteed to appear in the wave function regardless of their population. The *semistochastic approach* drastically reduces the stochastic error.^126,127^

### 2.2 The GAS Concept and the Stochastic-GAS Algorithm

The GAS concept^30^ is an extension and generalization of the *complete active space* (CAS) and the *restricted active space* (RAS)^128^ approaches. While in CAS and RAS, only one and three active subspaces are considered, respectively, the GAS concept allows for an arbitrary number of active subspaces, *k*. In RAS, the RAS1 and RAS3 subspaces consist of orbitals that are generally doubly occupied and empty, respectively. Users may decide on the maximum number of holes and particles allowed for the RAS1 and RAS3, correspondingly. The minimum number of holes and particles in RAS1 and RAS3 are always constrained to zero. In GAS, each subspace can contain occupied and empty orbitals alike, and minimum and maximum occupation numbers can be defined for each subspace by the user. *Intraspace excitations* are not restricted, i.e., in the generation of the GAS multiconfigurational wave function electrons are freely excited as long as they remain within their GAS subspace. On the contrary, *interspace excitations* are restricted by user-defined constraints. When interspace excitations are entirely forbidden the GAS subspaces are referred to as *disconnected*, when they are allowed the subspaces are referred to as *connected* (Figure 1).

**Figure 1:**
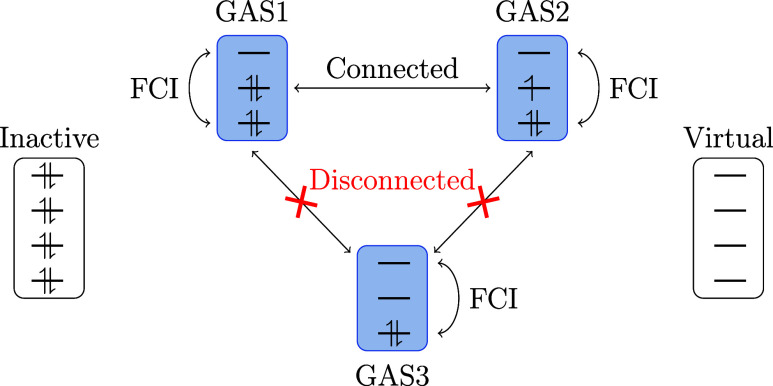
Graphical representation of a GAS space with three GAS subspaces. GAS1 and GAS2 are connected whereas GAS3 is disconnected.

For connected GAS subspaces, interspace excitations are generally defined either via *local* (eq 10) or *cumulative* (eq 11) constraints

10 $$\forall i,1\leq i\leq k:N_{i}^{\min}\leq x_{i}\leq N_{i}^{\max}$$

11 $$\forall i,1\leq i\leq k:{Ñ}_{i}^{\min}\leq \sum_{j=1}^{i}x_{j}\leq {Ñ}_{i}^{\max}$$

where *x*_*i*_ is the occupation number of the *i*–th subspace. Originally, the GAS algorithm used cumulative constraints, owing to its spin-adapted (GUGA) implementation.^30^ On the contrary, the ORMAS approach was implemented in the SD basis and local occupation number constraints were adopted.^32^ The different occupation constraints in ORMAS and the GAS, generally lead to different truncated CI expansions, as discussed in greater detail in the literature.^30^ However, the more recent Stochastic-GAS algorithm^31^ supports either occupation number constraints allowing to build both ORMAS and GAS truncated wave functions. Distributions of electrons among the GAS subspaces can be related to the mathematical concept of *compositions* ^31,129^ fulfilling the condition

12 $$x_{1}+x_{2}+\cdot \cdot \cdot +x_{k}=N,x_{i},N\in N_{0};k\in N$$

In the literature, compositions that fulfill the local/cumulative GAS constraints have been referred to as *supergroups*.^130,131^ Indices *i*_C_ and *i*_sg_ are assigned to compositions and supergroups, respectively, in decreasing lexicographic order. Table 1 illustrates the set of compositions and supergroups that are generated for a GAS space with three GAS subspaces (*k* = 3), a total of three electrons (*N* = 3) and local constraints {*N*_min_ = [0, 0, 1], *N*_max_ = [2, 2, 2]}. The equivalent set of cumulative constraints is defined as {*Ñ*_min_ = [0, 1, 3], *Ñ*_max_ = [2, 2, 3]}.

**Table 1:** Supergroup *i*_sg_ and Composition *i*_c_ Indices under GAS Constraints for 3 Electrons Distributed in 3 GAS Subspaces

*N*_min_		0	0	1
*N*_max_		2	2	1
*i*_sg_	*i*_C_	*x*_1_	*x*_2_	*x*_3_
	1	3	0	0
	2	2	1	0
**1**	**3**	**2**	**0**	**1**
	4	1	2	0
**2**	**5**	**1**	**1**	**1**
**3**	**6**	**1**	**0**	**2**
	7	0	3	0
**4**	**8**	**0**	**2**	**1**
**5**	**9**	**0**	**1**	**2**
	10	0	0	3

A GAS space is equivalent to the full CAS expansion if the set of valid supergroups {*i*_sg_} is equal to the set of compositions {*i*_C_}. This can be achieved by either selecting a single GAS space, or by selecting multiple GAS spaces, but allowing for all possible interspace excitations. For fully disconnected GAS subspaces, *N*_*i*_^min^ = *N*_*i*_^max^ (or analogously *Ñ*_*i*_^min^ = *Ñ*_*i*_^max^) one unique supergroup is permitted.

The notion of supergroups also allows the elegant implementation of the difference-dedicated CI (DDCI) algorithm.^132–135^ Therein, the active space is partitioned into three subspaces: a set of *n*_0_ doubly occupied inactive occupied orbitals, a set of *n*_a_ active orbitals containing *N*_a_ electrons, and a set of *n*_v_ inactive virtual orbitals, from here on referred to as GAS1, GAS2, and GAS3, respectively. GAS2 is a minimal valence CAS that offers a zeroth-order description of the underlying problem, e.g., the bonding and antibonding orbitals for a bond breaking process. This CAS space is extended by configurations stemming from excitations involving the outer GAS1 and GAS3 subspaces. These excitations are classified by the number of holes, *n*_h_, that are created in GAS1 and the number of particles, *m*_p_, that are created in GAS3. The DDCI space is defined to create all configurations that are achievable via up to double excitations among the subspaces, except for those that purely involve double excitations from GAS1 to GAS3, namely the 2*h* + 2*p* excitations. Allowed additional configurations are then those obtained via 1*h*, 1*p*, 1*h* + 1*p* (DDCI1, or CAS+S), 2*h*, 2*p* (DDCI2), 1*h* + 2*p*, and 2*h* + 1*p* excitations (DDCI, or DDCI3). It should be noted that every higher order DDCI space contains those of lower order, e.g., DDCI1 is contained in DDCI2. The full DDCI space is easily encoded into the supergroups by generating all supergroups provided by eq 13

13 $$\left[2\cdot n_{o}-n_{h},N_{a}+\left(n_{h}-m_{p}\right),m_{p}\right],n_{h},m_{p}\in \left\{1,2\right\},\;\left(n_{h},m_{p}\right)\neq \left(2,2\right)$$

Recently, we have proposed a Stochastic-GAS method,^31,136^ where the FCIQMC algorithm has been combined with the concepts of occupation number constraints and supergroups. In Stochastic-GAS, the FCIQMC dynamic is initiated from a reference configuration that fulfills the user-defined GAS specifications. GAS constraints are then ingrained at the excitation generation stage, i.e., excitations that violate GAS rules are *never* suggested. This step takes full advantage of the PCHB excitation generator. However, an excitation *AB* ← *IJ* can lead to GAS *forbidden* or *allowed* configurations depending on the parent configuration. As an example, we consider an active space model with three GAS subspaces, where orbitals *A* and *B* belong to GAS1, *I* to GAS2, and *J* to GAS3, constraining the occupation numbers to {*N*_min_ = [0, 0, 1], *N*_max_ = [2, 2, 2]}. A GAS allowed excitation is generated, if the excitation *A, B* ← *I, J* arises from the supergroup [0, 1, 2], whereas the same excitation is GAS forbidden, if it arises from the supergroup [1, 1, 1], as it exceeds the maximum number of particles in GAS1,

$$\underset{\mathrm{GAS}\;\mathrm{allowed}}{\underset{︸}{\left[2,0,1\right]}}\leftarrow \left[0,1,2\right]\underset{\mathrm{GAS}\;\mathrm{forbidden}}{\underset{︸}{\left[3,0,0\right]}}\leftarrow \left[1,1,1\right]$$

Hence, a configuration-independent probability that only depends on the excitation is no longer possible within GAS. Nonetheless, all configurations belonging to the same supergroup are equivalent in terms of excitation generation and it is possible to define PCHB weights for each supergroup. With these adjusted weights it is guaranteed that only GAS-allowed excitations are sampled for a given supergroup. Therefore, the supergroup of the starting configuration is the only required additional information to perform valid excitations on the basis of PCHB probability distributions. Determining the supergroup of a configuration is a very fast operation, which in practice means that there is zero run-time overhead to adhere to GAS constraints (see reference 31 for details). We use the previously introduced supergroup index *i*_sg_ and build supergroup-dependent PCHB probability tables, where PCHB weights representing GAS-forbidden excitations are set to zero

14 $${\mathrm{S̃}}_{I}^{A}\left(i_{\mathrm{sg}}\right)=\{\begin{array}{ll} S_{I}^{A} & \left(A\leftarrow I\right)\;\mathrm{GAS}\;\mathrm{allowed}\;\mathrm{for}\;i_{\mathrm{sg}}\\ 0 & \mathrm{else} \end{array}$$

15 $${\mathrm{W̃}}_{\mathrm{IJ}}^{\mathrm{AB}}\left(i_{\mathrm{sg}}\right)=\{\begin{array}{ll} W_{\mathrm{IJ}}^{\mathrm{AB}} & \left(AB\leftarrow IJ\right)\;\mathrm{GAS}\;\mathrm{allowed}\;\mathrm{for}\;i_{\mathrm{sg}}\\ 0 & \mathrm{else} \end{array}$$

These weights are used to calculate the underlying probability distribution for the FCIQMC procedure. Until now, GAS PCHB probabilities have been stored in dense data structures,^25,41,112^ that allow fast access to individual entries, under the assumption that entries in the PCHB tables are generally nonzero. For very flexible GAS models, with many GAS subspaces and varying interspace excitations, many supergroups are generated, and the memory demand for densely storing the PCHB tables becomes a bottleneck for large Stochastic-GAS computations, since their storage generally takes up the majority of the required memory. Moreover, according to eqs 14 and 15, the GAS PCHB probability tables present an exceedingly large number of zeroes. It is thus clear, that a dense representation of the GAS PCHB tables is less desirable and in some extreme cases prohibitively expensive. We envision that a *sparse representation* of the GAS PCHB probability tables is possible; details will be discussed in a separate document. Additionally, a numerical example is given in Section 3.5.

The GAS strategy allows the flexible modeling of chemical systems that can be described as ensembles of *spatially separated* (fragments) or *energetically separated* (shells) domains, in which the electronic structure of the ensemble arises from the interaction of the electronic structures of the domains. Polynuclear transition metal (PNTM) clusters, where the metal centers and the bridging ligands can be considered as separated domains, represent a prime example of spatially separated domains. In PNTM clusters, domains interact via spin-exchange mechanisms, metal-to-metal or ligand-to-metal hoppings. The latter form of interactions lead to the fascinating superexchange mechanism, that is at the core of the magnetic properties of these systems. Charge and energy transport in organic molecular crystals, relevant for singlet fission in organic photovoltaics, provide another example of spatially separated domains. These systems can be described as organized stacks of π-conjugated building blocks (domains), and the charge/energy transport occurs via the interactions of the π-electrons across the domains.^137–140^ In X-ray Absorption Spectroscopy (XAS), core orbitals and the outer shells form energetically separated domains, and their interactions via core-excitations lead to the formation of core-hole states.^141–146^ For all the above examples, the GAS concept can be utilized to identify the individual domains, whose electron correlation effects are accounted for by the intradomain excitations. Meanwhile, the user-defined interspace excitations describe the interdomain electron interactions.

In the context of perturbation theory, the GAS concept has already been utilized to construct highly flexible and chemically/physically relevant unperturbed multiconfigurational reference wave functions, followed by SplitGAS^111,147^ or GASPT2 ^81^ perturbative corrections. Stochastic-SplitGAS is a natural extension of uncontracted perturbative corrections within the stochastic framework.

### 2.3 Löwdin’s Partitioning Technique and the SplitGAS Method

Löwdin’s partitioning technique^110,148^ is a known perturbative method used to reduce the dimensionality of a large Hamiltonian matrix into a smaller one, while retaining the important spectral features of the original problem. To this end, the original space is partitioned into two subspaces, P and Q , where it is intended that dim(P)≪dim(Q) . The principal space, P , is thought to contain the configurations that are most critical to describe the qualitative electronic features of the underlying system. In turn, the perturber space, Q , is intended to contain the numerous additional configurations that are necessary for quantitative accuracy (dynamic correlation effects). The initial eigenvalue problem is mapped to a modified Hamiltonian, **H̃**, of dimensionality dim(P)^110,149^

16 $${\mathrm{H̃}}_{\mathrm{ij}}=H_{\mathrm{ij}}+\sum_{\alpha \in Q}\frac{H_{i\alpha }H_{\alpha j}}{E-H_{\alpha \alpha }}+\sum_{\alpha ,\beta \in Q}\frac{H_{i\alpha }H_{\alpha \beta }H_{\beta j}}{\left(E-H_{\alpha \alpha }\right)\left(E-H_{\beta \beta }\right)}+\cdot \cdot \cdot$$

The partitioning into P and Q may be defined by a wide array of criteria such as occupation number or energy,^149^ excitation levels with respect to a single electronic configuration for example the Hartree–Fock configuration,^150^ or schemes like CIPSI that progressively add determinants to the P space.^151–153^ Previous efforts include methods such as SplitCAS,^149^ Bk^150^ and shifted-Bk.^46,154–157^ This partitioning strategy has also been successfully used in coupled cluster (CC) theory to approximate higher-order CC methods^158–160^ or CC in a larger active space at a fraction of the cost.^161^ In 2013, a strategy has been proposed where the separation into the P and Q classes is achieved by defining two disjoint GAS spaces, from which the name of the method, SplitGAS, originates.^111^ In SplitGAS, eq 16 is truncated after the second term, for computational efficiency reasons that will be discussed in the next section. The SplitGAS method has been demonstrated to be a powerful tool to describe the electronic structure of complex electronic structures for which both strong and dynamic correlation effects are important. For example, the method could simulate the dissociation of the Cr_2_ dimer, often considered as a benchmark for the performance of quantum chemical methods.^111^ The good agreement between the SplitGAS and the experimental data, both in terms of dissociation energy, equilibrium bond length and vibrational frequencies, has motivated us to use the same paradigm for even larger systems. Nonetheless, the SplitGAS­(12,12)//(24,48) space, with a CAS­(12,12) P -space, utilized for the Cr_2_ system is already at the limit of what can be achieved with the conventional/nonstochastic method, preventing the application of SplitGAS to systems requiring even larger active spaces, in P or in the overall P+Q spaces. The experience accumulated in combining the FCIQMC algorithm with the CAS^28^ and GAS^31,136^ concepts has led us to the development of a stochastic version of SplitGAS, which is the main focus of this work, whose algorithm is described in the next section.

### 2.4 Stochastic-SplitGAS Algorithm

This section describes the algorithmic details of Stochastic-SplitGAS. The SplitGAS main working equation is based on the general form of Löwdin’s equation truncated to the second-order term

17 $${\mathrm{H̃}}_{\mathrm{ij}}=H_{\mathrm{ij}}+\sum_{\alpha \in Q}\frac{H_{i\alpha }H_{\alpha j}}{E-H_{\alpha \alpha }}$$

The elements needed to construct a specific entry of the downfolded Hamiltonian, *H̃*_*ij*_, are the *H*_*ij*_ matrix element in the PP block of the original Hamiltonian, the connecting matrix elements *H*_*i*α_ and *H*_α*j*_ in PQ and QP to every configuration |ϕ_α_⟩ in Q that can be reached from |ϕ_*i*_⟩ and |ϕ_*j*_⟩ and the corresponding diagonal elements *H*_αα_ in the QQ block (see Figure 2). None of the off-diagonal terms of the QQ block are necessary in building the SplitGAS downfolded Hamiltonian. The SplitGAS downfolded Hamiltonian in the PP block is equivalent to an unfolded Hamiltonian that spans the P and Q partitions, but with off-diagonal elements in the QQ block set to zero (see Appendix [Sec app1-sec1] for more details).

The unfolded form of the modified Hamiltonian with a diagonal QQ block, in the following referred to as *effective* Hamiltonian (see Figure 2), retains the dimensionality dim(P+Q) of the original Hamiltonian, and thus also the same challenges as the full eigenvalue problem if treated conventionally. For this reason the folding procedure was preferred in the conventional SplitGAS approach.^111^ However, FCIQMC takes advantage of sparsity in the Hamiltonian and in virtue of the reduced couplings (all off-diagonal entries in the QQ block are set to zero), the stochastic exploration of the *effective* SplitGAS Hamiltonian and its resolution via the FCIQMC imaginary-time propagation features a higher efficiency as compared to the original Hamiltonian (vide infra). The stochastic exploration of a downfolded instead of the proposed *effective* Hamiltonian faces two immediate challenges. First, we generally expect the downfolding procedure to lead to a denser matrix **H̃**. Second, the modified matrix elements, eq 17, used for the spawning and death steps of the FCIQMC algorithm are to be calculated on-the-fly at each imaginary-time step, adding an impractical computational overhead.

**Figure 2:**
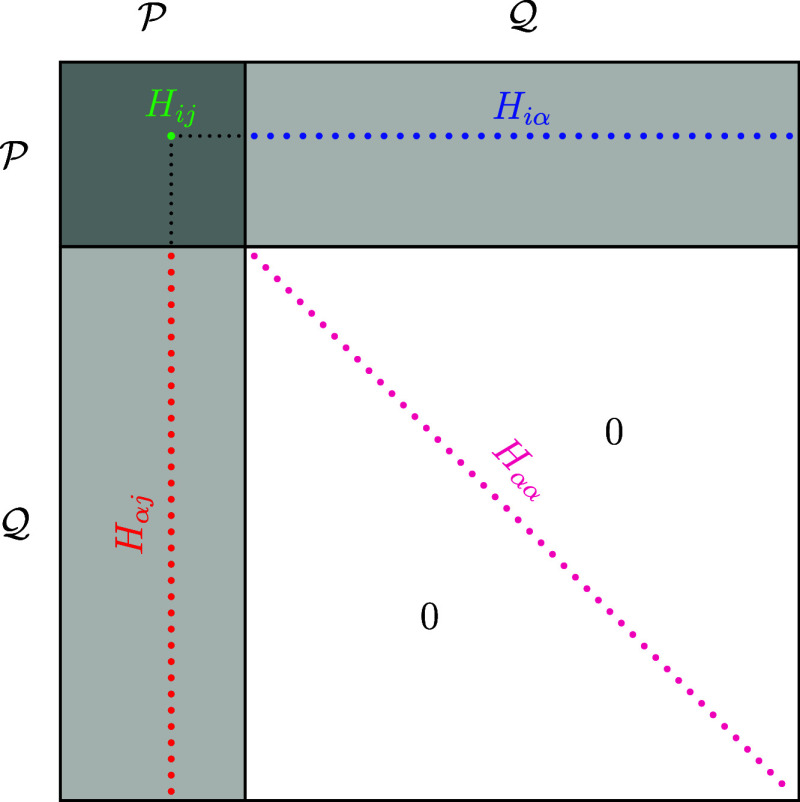
Schematic structure of the effective SplitGAS Hamiltonian matrix.

Using the *effective* rather than the *downfolded* Hamiltonian avoids both the need to select the energy term *E*, appearing in the denominator of eq 17, and the iterative procedure required to converge it. In the downfolded Hamiltonian, the trial energy *E* is typically chosen as the ground-state energy of the unperturbed P space (though excited states can also be targeted). Therefore, as the downfolded Hamiltonian depends explicitly on *E*, it is only possible to target one electronic state at a time.^162,163^ By eliminating both the selection and iterative optimization of *E*, Stochastic-SplitGAS enables a less biased simultaneous treatment of multiple electronic states.

*Based on the existing FCIQMC algorithm, how do we design an efficient Stochastic-SplitGAS that only explores the coupling terms in* PP , PQ , *and* QP *but not the off-diagonal in* QQ *?*

We are presented with the same challenge as for the Stochastic-GAS approach, where from an efficiency standpoint, excitations should not be generated, checked, and discarded on the basis of violation rules (either GAS-forbidden electron distributions, or off-diagonal elements within the QQ block). While this strategy is feasible, it is inefficient and defeats the purpose of exploring and solving a simplified effective Hamiltonian. Instead, similarly to our earlier Stochastic-GAS algorithm, all required constraints must be encoded in the excitation generation step, in a way that no excitations leading to off-diagonal QQ elements are ever suggested at the *excitation generation* stage.

The P and Q spaces are defined by two disjoint sets of *supergroups*, $\left\{i_{\mathrm{sg}}^{P}\right\}\cap \left\{i_{\mathrm{sg}}^{Q}\right\}=⌀$ . From an algorithmic point of view, we introduce the union T=P∪Q , and create a logical vector $\left\{i_{P}\right\}$ where supergroups in P are denoted as true and all remaining supergroups as false, thus, defining the Q space. The structure of $\left\{i_{P}\right\}$ is illustrated in Table 2.

**Table 2:** Connection between Supergroups of T and P , and the Logical Vector $\left\{i_{P}\right\}$

*i*_C_	$i_{\mathrm{sg}}^{T}$	*i*_sg_^*P*^	$i_{P}$
1	–	–	–
2	1	–	false
3	–	–	–
4	2	1	true
5	3	–	false
6	–	–	–
7	4	–	false
8	5	2	true
⋮	⋮	⋮	⋮

Compositions *i*_C_ that are not valid supergroups of P or Q are not part of the logical vector $\left\{i_{P}\right\}$ .

For any spawning event, the starting and the target configurations, $i_{P}$ and $i_{P}^{\prime }$ , respectively, are drawn from the $\left\{i_{P}\right\}$ logical vector, with four possible outcomes

$$\left|\phi^{\prime }\right\rangle \leftarrow \left|\phi \right\rangle =\{\begin{array}{l} i_{\mathrm{sg}}^{\prime }\in \left\{i_{\mathrm{sg}}^{P}\right\}\leftarrow i_{\mathrm{sg}}\in \{i_{\mathrm{sg}}^{P}\}\;\mathrm{or}\;P\leftarrow P\\ i_{\mathrm{sg}}^{\prime }\notin \left\{i_{\mathrm{sg}}^{P}\right\}\leftarrow i_{\mathrm{sg}}\in \{i_{\mathrm{sg}}^{P}\}\;\mathrm{or}\;Q\leftarrow P\\ i_{\mathrm{sg}}^{\prime }\in \left\{i_{\mathrm{sg}}^{P}\right\}\leftarrow i_{\mathrm{sg}}\notin \{i_{\mathrm{sg}}^{P}\}\;\mathrm{or}\;P\leftarrow Q\\ i_{\mathrm{sg}}^{\prime }\notin \left\{i_{\mathrm{sg}}^{P}\right\}\leftarrow i_{\mathrm{sg}}\notin \{i_{\mathrm{sg}}^{P}\}\;\mathrm{or}\;Q\leftarrow Q \end{array}$$ 18

All off-diagonal elements in the QQ block of the SplitGAS Hamiltonian are zero (Figure 2). As a result, within FCIQMC, any excitation that connects configurations of the QQ block (last case in eq 18) must have zero acceptance probability, *p*_acc_ = 0. Such excitations should therefore be excluded from spawning. Inspection of the possible cases in eq 18 and their mapping to the logical vector $\left\{i_{P}\right\}$ of Table 2, show that exclusion of intra- Q excitations can promptly be identified by applying an OR-Gate to the respective entries in $\left\{i_{P}\right\}$ , as shown in Table 3.

**Table 3:** Result of the OR-Gate (∨) on the Entries in the $\left\{i_{P}\right\}$ Logical Vector

case	$i_{P}^{\prime }$	$i_{P}$	$i_{P}^{\prime }\vee i_{P}$
P←P	true	true	true
Q←P	false	true	true
P←Q	true	false	true
Q←Q	false	false	false

In practice for a given supergroup, *i*_sg_, (a) the *A, B* ← *I, J* orbital indices identify the target supergroup, *i*_sg_^′^, (b) the OR-Gate singles out *Q* ← *Q* excitations via $\left(i_{P}^{\prime }\vee i_{P}\right)$ = false, and (c) the corresponding entry in the PCHB table is set to zero (see eq 19).

$$\begin{array}{l} {\mathrm{S̃}}_{I}^{A}\left(i_{\mathrm{sg}}\right)=\{\begin{array}{ll} \{\begin{array}{ll} S_{I}^{A} & \mathrm{if}\;i_{P}^{\prime }\vee i_{P}\\ 0 & \mathrm{if}\neg \left(i_{P}^{\prime }\vee i_{P}\right) \end{array} & \left(A\leftarrow I\right)\;\mathrm{GAS}\;\mathrm{allowed}\\ 0 & \mathrm{else} \end{array}\\ {\mathrm{W̃}}_{\mathrm{IJ}}^{\mathrm{AB}}\left(i_{\mathrm{sg}}\right)=\{\begin{array}{ll} \{\begin{array}{ll} W_{\mathrm{IJ}}^{\mathrm{AB}} & \mathrm{if}\;i_{P}^{\prime }\vee i_{P}\\ 0 & \mathrm{if}\neg \left(i_{P}^{\prime }\vee i_{P}\right) \end{array} & \left(AB\leftarrow IJ\right)\;\mathrm{GAS}\;\mathrm{allowed}\\ 0 & \mathrm{else} \end{array} \end{array}$$ 19

This strategy allows efficient generation and access to the PCHB weights corresponding to the SplitGAS effective Hamiltonian within FCIQMC. Thus, referring to eq 19, the weight for the *AB* ← *IJ* spawning event will correspond to the original *W*_*IJ*_^*AB*^ value, if it corresponds to any element of the PP , PQ and QP blocks of the GAS allowed configurational space, and to zero if it corresponds to a GAS forbidden configuration or to an off-diagonal element of the QQ block. The outlined algorithm highlights the advantage of SplitGAS partitioning over a selection scheme such as CIPSI. Although a (hypothetical) CIPSI-like selection of the P space within FCIQMC might be more fine-grained and of higher-quality, the notion of supergroups no longer exists. Hence, a per configuration encoding within the excitation generator would be required, thereby making it intractable within a stochastic framework.

Stochastic-SplitGAS has also been adapted to the *semistochastic* part of the dynamics, which performs deterministic, imaginary-time propagation of important selected configurations (core space), namely

$$H_{\mathrm{ij}}^{\mathrm{core}}=\{\begin{array}{ll} \{\begin{array}{ll} \left\langle \phi_{i}|Ĥ|\phi_{j}\right\rangle & \mathrm{if}\;i_{P}^{\prime }\vee i_{P}\\ 0 & \mathrm{if}\neg \left(i_{P}^{\prime }\vee i_{P}\right) \end{array} & |\phi_{i}\rangle \;\mathrm{and}\;|\phi_{j}\rangle \;\mathrm{GAS}\;\mathrm{allowed}\\ 0 & \mathrm{else} \end{array}$$ 20

A stochastic implementation of Löwdin downfolding was previously developed by Ten-no and co-workers, in the context of the model space quantum Monte Carlo (MSQMC) strategy.^164^ In contrast to the implementation presented here, a transfer matrix $T_{\mathrm{QP}}$ is sampled stochastically which can be used to determine the downfolded effective Hamiltonian as well as the CI coefficients in Q . MSQMC has the advantage that the working equation (eq 16) is not truncated after the second term, resulting in a higher quality perturbation, which incidentally should also avoid spin-contamination (as discussed in Appendix A.2). In addition, excited states can be specifically optimized by selecting the parameter *E* accordingly, and a state-selective partitioning allows several solutions to be obtained simultaneously at the price of one.^165^ However, the dependence of the result on the energy *E* has two main disadvantages, namely numerically different results of varying quality can be obtained for a system depending on how *E* is chosen, and the solution must be iteratively converged (*E* update). These limitations are reflected in the scalability of the method as the largest calculation ever performed, to the best of our knowledge, correlated 24 electrons in 30 orbitals.^165^

Stochastic-SplitGAS is able to simultaneously optimize multiple electronic states by enforcing orthogonality among the wave functions.^166^ The PCHB table is shared among the electronic states, and the added computational cost is merely that of storing the walker population of multiple wave functions. Furthermore, it is also possible to target states belonging to the abelian point group *D*_2h_, or any of its subgroups. Stochastic-SplitGAS is expected to be less susceptible to the Fermionic sign problem^24,167^ than a Stochastic-GAS calculation in the respective T space, due to a reduction of spawns that could promote sign instabilities given the diagonal approximation in QQ .

### 2.5 SplitGAS for Slater Determinant and Spin-Adapted Bases

In the exact (nonrelativistic) FCI framework without magnetic fields, working in SD or spin-adapted basis is equivalent: the Hamiltonian commutes with *S̃* ^2^, yielding spin-pure eigenstates. This is not necessarily true for the downfolded Hamiltonian. We have implemented the Stochastic-SplitGAS both for SD and spin-adapted (GUGA) bases. It is thus relevant to discuss the differences between the two variants. A small CAS­(2,2) space, T , is considered as an example, restricting the spin-adapted basis to the singlet spin multiplicity (*S*_tot_ = 0) and the space of SDs to elements with zero total spin projection (*M*_*S*_ = 0). The sets of SDs and CSFs are listed in eqs 21 and 22, in that order. In the CSF basis, doubly occupied and empty orbitals are labeled as 2 and 0, while *u* and *d* denote singly occupied orbitals with cumulative spin-up and spin-down coupled electrons, respectively. Eq 22 is further expanded into its SD components in eq 23 to allow for a direct comparison with eq 21.

23 TSD={|2,0⟩,|α,β⟩,|β,α⟩,|0,2⟩}(21)TGUGA={|2,0⟩,|u,d⟩,|0,2⟩}(22)≡{|2,0⟩,1/2(|α,β⟩−|β,α⟩),|0,2⟩}(23)

A single configuration is chosen for the P space for both many-electron bases, namely

24 P={|2,0⟩}→H=[⟨2,0|Ĥ|2,0⟩]

After the downfolding (eq 17), the modified Hamiltonian matrix element in the two bases reads as

$${\mathrm{H̃}}_{11}^{\mathrm{SD}}=\left\langle 20\right|Ĥ\left|20\right\rangle +\frac{|\left\langle 20\right|Ĥ|02\rangle {|}^{2}}{E_{0}-\left\langle 02\right|Ĥ\left|02\right\rangle }+\frac{|\left\langle 20\right|Ĥ|\alpha \beta \rangle {|}^{2}}{E_{0}-\left\langle \alpha \beta \right|Ĥ\left|\alpha \beta \right\rangle }+\frac{|\left\langle 20\right|Ĥ|\beta \alpha \rangle {|}^{2}}{E_{0}-\left\langle \beta \alpha \right|Ĥ\left|\beta \alpha \right\rangle }$$ 25

26 $${\mathrm{H̃}}_{11}^{\mathrm{CSF}}=\left\langle 20\right|Ĥ\left|20\right\rangle +\frac{|\left\langle 20\right|Ĥ|02\rangle {|}^{2}}{E_{0}-\left\langle 02\right|Ĥ\left|02\right\rangle }+\frac{\frac{1}{2}\left|\left\langle 20\right|Ĥ\right|\alpha \beta \rangle {|}^{2}+\frac{1}{2}\left|\left\langle 20\right|Ĥ\right|\beta \alpha \rangle {|}^{2}-\left\langle 20\right|Ĥ\left|\alpha \beta \right\rangle \left\langle 20\right|Ĥ\left|\beta \alpha \right\rangle }{E_{0}-\frac{1}{2}\left\langle \alpha \beta \right|Ĥ\left|\alpha \beta \right\rangle -\frac{1}{2}\left\langle \beta \alpha \right|Ĥ\left|\beta \alpha \right\rangle +\left\langle \alpha \beta \right|Ĥ\left|\beta \alpha \right\rangle }$$

The first two terms of the above equations, corresponding to pure singlet terms, are identical for the two bases, while the remaining terms differ. A direct comparison of the configurations with unpaired electrons in SD basis ({|αβ⟩, |βα⟩}) and spin-adapted basis ({1/2(|αβ⟩−|βα⟩)}) shows that the difference between the two effective Hamiltonian matrices arises from the spin adaptation constraint in the latter basis. Thus, within the SplitGAS framework, the equivalence in the spectra and properties obtained in the two bases breaks down. The unconstrained downfolding within the SD basis yields a *spin contaminated* perturbative correction, potentially leading to unphysical eigensolutions, even if the zeroth-order wave function (in P ) is a pure spin state. On the contrary, the constrained downfolding within the spin-adapted basis prevents any spin-contaminated perturbative correction. An algebraic and numerical analysis of the contamination is detailed in Appendix A.2. This aspect is especially relevant in the context of exchange-coupled magnetic systems, where the spin contamination of the perturber space can cause the artificial splitting (or mixing) of spin states, while breaking spin symmetry.

Another feature that differentiates the two bases is the sparsity of the corresponding Hamiltonian matrices. An increased sparsity in the original QQ block is expected to lead to a more accurate perturbative correction as the effective Hamiltonian is more similar to the full Hamiltonian. One way to achieve this is through orbital transformations such as localization. Recent findings based on *Quantum Anamorphosis* ^168^ suggest that in spin-adapted bases the Hamiltonian matrix may assume a unique block-diagonal structure, (both in P and Q spaces) via specific molecular orbital (MO) transformations in the form of localization and reordering.^29,69,70,169–172^

## 3 Applications

Three applications of Stochastic-SplitGAS are presented to numerically demonstrate the robustness of the method and importantly its scalability. In the first validation example, nonstochastic calculations are performed within a small CAS­(12,9) active space for the ozone system, to compare the SplitGAS wave function to the available exact wave function on the same orbital space. In the second example, we demonstrate the scalability of Stochastic-SplitGAS on an Fe­(II)-porphyrin model system, already studied by some of us in other works.^31,68,136,173,174^ For this system, the Stochastic-SplitGAS P -space is generated from a large CAS­(32,34) active space, while the perturber Q -space spans a total of 96 electrons and 159 orbitals, corresponding to the space chosen for a Stochastic-MRCI calculation in reference 31. The strict comparison between Stochastic-SplitGAS and Stochastic-MRCI is particularly relevant to assess the performance of the perturbative approach, while reducing the computational cost. In the last example, we study the relative stability of the energetically low-lying spin states (spin ladder) of a [Fe­(III)_2_S_2_]^2–^ ferredoxin model complex. The example is designed to test the applicability of the Stochastic-SplitGAS method in the context of magnetic systems dominated by exchange interactions across transition metal centers, mediated by bridging ligands, which are generally considered an important challenge for many electronic structure methods. The superexchange mechanism, which is at the core of the relative stability of the low-energy spin states, is explicitly described within the larger CAS­(22,26) active space. *Can it be correctly described by the less expensive Stochastic-SplitGAS method?*

### 3.1 Computational Details

All necessary steps, including CASSCF, localization, and the generation of the FCIDUMP files were performed within OpenMolcas.^175^ All calculations were performed in C_1_ point-group symmetry. Stochastic-SplitGAS has been implemented within the NECI program,^25^ and all stochastic calculations were performed on that code. The initiator approximation^27^ was used for all our dynamics, to reduce the Fermionic sign problem bias. The GUGA basis was employed for all test case applications unless specified otherwise. The spin ladder of [Fe­(III)_2_S_2_]^2–^ and the ozone singlet–triplet spin gap were computed in both the GUGA and SD basis, to gain insight into the influence of the chosen basis onto both the perturbative corrections and on spin contamination effects. For all stochastic simulations, the standard error was estimated either via blocking analysis^176,177^ of the data or, for properties derived from RDMs sampled at intervals exceeding the autocorrelation length, by direct computation of the standard error.

SplitGAS calculations are denoted as P ­[···], where the P space is defined within brackets. Furthermore, the short notation CAS­(*e, o*) + (*a, b, c*) was chosen, where CAS­(*e, o*) refers to the minimal P space, and *a*, *b*, *c* are the maximum number of particles that can be excited into or out of GAS1, GAS2, and GAS3, respectively, mimicking an MRCI-type expansion of the P space. Notably, the flexibility of the GAS concept is such that P and Q spaces can be defined in a variety of ways, depending on the system and the desired accuracy, beyond the particular choices made for the examples presented here, which are limited to MRCI-type expansions.

#### Ozone

For the lowest singlet spin state of the ozone molecule,^178,179^ the CASSCF­(12,9) natural orbitals, using an ANO-RCC-VDZP^180^ basis set, were used, consisting of the nine 2p orbitals on the oxygen atoms and their 12 electrons. The three π-type orbitals were localized using Pipek–Mezey^181^ into the three nearly degenerate p_*z*_ atomic-like orbitals, for a subsequent rationalization of the leading configurations contributing to the ground state wave function. Orbitals were sorted as 3·p_*z*_, 4·σ, and 2·σ*, to maximize sparsity within the GUGA basis.^29,69,168–172^ We defined three GAS subspaces containing the p_*z*_, σ and σ* orbitals, respectively. The local occupation number constraints of the total, T , and the principal space, P , are listed in Table 4.

**Table 4:** Local GAS Occupation Number Constraints of the P and T Space for Ozone

P [CAS(4,3) + (1,1,2)]					
		T		P	
*i*	*N*_*i*_^orb^	*N*_*i*_^min^	*N*_*i*_^max^	*N*_*i*_^min^	*N*_*i*_^max^
G1 (p_*z*_)	3	0	6	3	5
G2 (σ)	4	0	8	7	8
G3 (σ*)	2	0	4	0	2

All possible interspace excitations within T have been allowed, making it equivalent to the CASCI­(12,9) configurational space. The P space was constrained to single excitations out of the G2 space and up to double excitations into the G3 space, while allowing the occupation of the G1 space to range between 3 and 5 (with one electron more/less with respect to 4, which is the accepted occupation number of the π-type orbitals for this system). Given the limited size of the T space, all calculations were performed deterministically using proprietary python pilot code.^182^ The total CAS­(12,9) wave function and its energy were used as reference values, to which the wave function and energies of approximated methods were compared to, namely P -space-only and SplitGAS calculations. The ⟨*Ŝ* ^2^⟩ expectation values were calculated deterministically in a SD basis via Stochastic-SplitGAS (semistochastic approximation across all configurations) for the aforementioned and a minimal CAS­(4,3) P space.

#### Fe­(II)-Porphyrin Model System

Calculations were performed for the ^3^E_g_ and the ^5^A_1g_ states of a model system that we introduced and used in a number of earlier works,^31,68,136,173^ where the β-carbon atoms have been removed and saturated with hydrogen atoms. The Stochastic-CASSCF­(32,34)^68^ natural orbitals, within the ANO-RCC-VTZP basis set,^183,184^ were utilized for this system, for generating the P space. The active space consisted of nine doubly occupied π, seven empty π*, five metal centered 3d orbitals and their 6 electrons, four doubly occupied σ_N_, four empty orbitals of the 4s4p shell, and five empty correlating d’ orbitals. The active orbitals were localized via Pipek–Mezey, and separated in two groups, (i) localized orbitals with high Mulliken population on the metal center, and (ii) the remaining localized orbitals, mostly with 2p_*z*_ character of the carbon atoms of the macrocycle. The one-body density matrices for the two orbital subsets were built and diagonalized separately, leading to fragment separated pseudonatural orbitals. A Procrustes transformation^185^ of the orbitals on the metal center was performed to achieve maximum similarity between the molecular orbitals on the metal and the metal centered atomic orbitals. The localization/Procrustes procedure was enforced to facilitate the rationalization of the CAS­(32,34) wave function. The resulting orbitals are depicted in reference 136. The Q space was built in analogy to the Stochastic-GAS­(96,159) from reference 136; 32 doubly occupied orbitals were chosen in a GAS1 subspace, leaving only 21 core orbitals uncorrelated (frozen), and 93 virtual orbitals above the CAS­(32,34) were added to a GAS3 subspace. In reference 136, an uncontracted Stochastic-MRCI­(SD) was performed by considering the CAS­(32,34) as multiconfigurational reference space, while outer correlation was added via single and double excitations from the GAS1 and to the GAS3 spaces. In the context of Stochastic-SplitGAS, rather than fully correlating the GAS­(96,159) space, all single and double excitations from the GAS1 space, and into the GAS3 space form the Q space and are treated perturbatively (Table 5).

**Table 5:** Cumulative GAS Constraints of the P and T Space for Fe­(II)-Porphyrin

P [CAS(32,34)]					
		T		P	
*i*	*N*_*i*_^orb^	*Ñ*_*i*_^min^	*Ñ*_*i*_^max^	*Ñ*_*i*_^min^	*Ñ*_*i*_^max^
G1	32	62	64	64	64
G2	34	94	96	96	96
G3	93	96	96	96	96

The adaptive-shift method was employed for these calculations,^186,187^ in line with the Stochastic-MRCI­(SD) calculations of reference 136. The calculations were converged for a walker population *N*_w_ = 5·10^8^. Inputs for the singlet and triplet can be found in Section SI.S1.

#### [Fe­(III)_2_S_2_]^2–^ Model System

A CAS­(22,26) active space was chosen for this system,^169^ consisting of the six doubly occupied 3p orbitals on the bridging sulfur ligands, the ten valence 3d molecular orbitals of the two magnetic centers with their 10 electrons, and the ten empty correlating d’ orbitals on the iron centers. The variationally optimized spin-adapted Stochastic-CASSCF­(22,26) natural orbitals for each spin-state,^29^ were used for performing the Stochastic-SplitGAS calculations. The corresponding Stochastic-CASSCF­(22,26) energies are used as a reference. A CASSCF­(10,10) within the CAS­(22,26) was performed, consisting solely of the 3d orbitals and their unpaired electrons, aiming at maximizing electron correlation within the magnetic orbitals. Subsequent CASPT2 calculations were performed using the CASSCF­(10,10) wave function as reference, and exclusively within the CAS­(22,26), allowing us to directly compare CASPT2 to the CAS­(22,26) reference. The ANO-RCC-VDZ basis was chosen for the metal centers, and the ANO-RCC-MB for all remaining atoms. The two-electron integrals were Cholesky decomposed with a decomposition threshold of 1·10^–6^.^188–190^

For the SplitGAS calculations, the 3d and d′ orbitals were further localized separately following the Pipek–Mezey procedure, while the 3p orbitals were kept delocalized across the two sulfur bridging ligands. In order to maximize correlation within the P space and sparsity within the Q space, the 26 active orbitals were site ordered as 5·3d_*A*_, 5·d_*A*_^′^, 5·3d_*B*_, 5·d_*B*_^′^, and 6·3p_S_, where *A* and *B* refers to the two iron centers, and the last six orbitals are the delocalized 3p orbitals of the bridging sulfur ligands. Three GAS subspaces were generated, consisting of (a) the localized 3d orbitals over the two metal centers (GAS1), (b) the localized d’ (GAS2), and (c) the delocalized six 3p orbitals of the ligands (GAS3). Three different Stochastic-SplitGAS setups were performed, one with the P space consisting of the full-CI expansion exclusively within the GAS1 subspace, one where the P space also includes single excitations from GAS3 and into GAS2, and one where the P space includes up to double excitations from GAS3 and into GAS2. Single and double excitations out of the configurations of the P space form the Q space (Table 6).

**Table 6:** Local GAS Constraints of the P and T Spaces for the [Fe­(III)_2_S_2_]^2–^ System

P [CAS(10,10)]					
		T		P	
*i*	*N*_*i*_^orb^	*N*_*i*_^min^	*N*_*i*_^max^	*N*_*i*_^min^	*N*_*i*_^max^
G1	10	8	12	10	10
G2	10	0	2	0	0
G3	6	10	12	12	12

P [CAS(10,10) + (1,1,1)]					
		T		P	
*i*	*N*_*i*_^orb^	*N*_*i*_^min^	*N*_*i*_^max^	*N*_*i*_^min^	*N*_*i*_^max^
G1	10	7	13	9	11
G2	10	0	3	0	1
G3	6	9	12	11	12

P [CAS(10,10) + (2,2,2)]					
		T		P	
*i*	*N*_*i*_^orb^	*N*_*i*_^min^	*N*_*i*_^max^	*N*_*i*_^min^	*N*_*i*_^max^
G1	10	6	14	8	12
G2	10	0	4	0	2
G3	6	8	12	10	12

Exemplary GAS and SplitGAS inputs and details about the respective walker populations can be found in Section SI.S1.

A SD basis was employed in a P ­[CAS­(10,10) + (1,1,1)] space to analyze the spin contamination effects. Therein, the ⟨*Ŝ* ^2^⟩ expectation values of the spin ladder were calculated with a population of 5 million walkers.

### 3.2 Results and Discussion

#### 3.2.1 Ozone Wave Function

Figure 3 shows the CI coefficients of the electronic ground-state wave function obtained from the exact diagonalization of the T space, as well as the corresponding deviations, Δ*c*(*i*), when solving exclusively the P space or the SplitGAS Hamiltonian. The coefficients belonging to P and Q are sorted into two regions that are separated via the green dashed line. Additionally the RMSE of the SplitGAS and P space wave functions with respect to the entire T space wave function are reported. No particular order for the configuration index, *i*, was followed except the partitioning of configurations in the P and the Q spaces.

**Figure 3:**
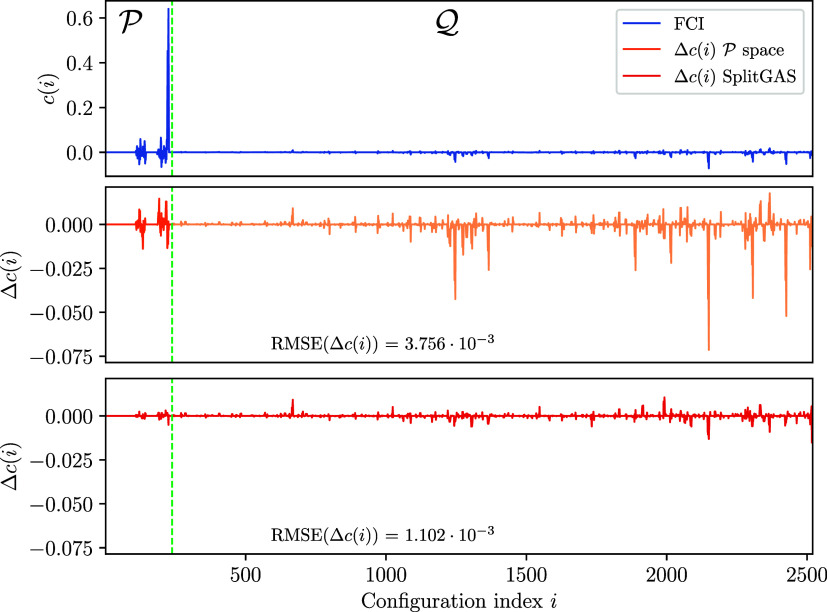
CI coefficients of the exact CAS­(12,9) wave function (in the GUGA spin-adapted basis) for the lowest singlet spin state of the ozone system, and the deviations obtained when solving for the P space and SplitGAS wave functions.

The wave function obtained via P diagonalization has zero components from the Q space, with obvious 100% deviation from the exact T space diagonalization in that space. However, also the deviations in the P space are substantial. In contrast, albeit approximated, the SplitGAS wave function has components both in P and Q spaces, and a substantial portion of the exact wave function is recovered, as demonstrated by the considerable reduction of the error, both in P and Q . This result suggests that SplitGAS is capable of enhancing the entire wave function in the total space, at a much reduced computational cost, in virtue of the diagonal approximation in the QQ block. This result is also reflected in the total energies. The P space ground state energy is 62.7 mHa higher, whereas SplitGAS only 5.3 mHa higher than the CAS­(12,9) reference energy (−224.725 236 Ha).

The ⟨*Ŝ* ^2^⟩ expectation values in the SD basis exhibit significant spin-contamination (see Table 9 in Appendix [Sec app1-sec2]).

#### 3.2.2 Fe­(II)-Porphyrin Model System

The intricate interplay of ligand field and many-body correlation interactions between the metal center and the large macrocycle, makes predictions of the relative stability of the near-degenerate low-energy quintet (^5^A_1g_) and triplet (^3^E_g_) spin states of the Fe­(II)-porphyrin system a significant theoretical challenge, and historically this system has been regarded as a test case to assess the effectiveness of novel electronic structure methods. There is general consensus that the triplet is the ground state of the parent Fe­(II)-porphyrin system, which is also supported by experimental data.^191–194^ Using a CAS­(14,16) reference wave function, consisting of the five 3d valence orbitals and their six electrons, the five empty d’ correlating orbitals, the doubly occupied σ orbital (of same symmetry as the $d_{x^{2}-y^{2}}\;(e_{g})$ orbital), the doubly occupied semicore 3s orbital, and the four Gouterman orbitals^195^ of the macrocycle (two occupied and two unoccupied frontier orbitals), followed by the well-established conventional contracted second-order perturbation theory, CASPT2, erroneously predicts a quintet ground state.^68^ Expanding the active space to the entire 16 π orbitals of the macrocycle with their 18 electrons, the empty 4s4p shell at the metal center, the additional three symmetry-adapted doubly occupied orbitals at the nitrogen atoms (with 2p_*x*_ and 2p_*y*_ contributions), CAS­(32,34) (the semicore 3s orbital was removed from this model active space) predicts the correct triplet ground state, with the quintet at 3.5 kcal/mol above.^68^

Accounting for dynamic correlation effects outside the large CAS­(32,34) represents an important methodological challenge. Recently, we have developed a contracted Stochastic-CASPT2 approach, based on time-averaging of the stochastic wave function.^105^ Due to the rapid growth of the necessary time-averaged wave function with the active space size, converging the four-body RDMs contracted with the Fock matrix for the CAS­(32,34) reference was computationally prohibitive. At the reduced Stochastic-CASSCF­(26,27) level, the quintet remains the ground state of the system, with the triplet lying 8.8 kcal/mol higher, in stark contrast to the Stochastic-CASSCF­(32,34) result. The subsequent Stochastic-CASPT2­(26,27) calculation reduces the gap to 2.2 kcal/mol, but still favors the quintet spin state, despite accounting for dynamic correlation effects.

Recent efforts by Neese et al. have led to a new FR-NEVPT2 algorithm that is applicable to systems with large active spaces via approximate density matrices.^90^ This new algorithm has been applied to the Fe­(II)-porphyrin system with up to a CAS­(30,29) reference space. Even in a QZ basis, such large active space FR-NEVPT2 strategy predicts a quintet ground state with a gap of about 7.7 kcal/mol.

In a separate work, we further expanded the CAS­(32,34) active space to explicitly include semicore correlation from the metal-centered 3s3p shell, resulting in a CAS­(40,38) description. The ^5^A_1g_–^3^E_g_ gap increases to 4.4 kcal/mol, indicating that correlation effects beyond CAS­(32,34) further stabilize the triplet spin state.^173^ To that end, as part of our Stochastic-GAS development, we also devised an uncontracted Stochastic-MRCI­(SD) approach, using the CAS­(32,34) as the reference wave function. The MRCI wave function was expanded by all single and double excitations within a significantly larger GAS­(96,159) space, yielding a ^5^A_1g_–^3^E_g_ gap of 7.0 kcal/mol, twice the value obtained from the bare Stochastic-CASSCF­(32,34).^31^ Larger active spaces, such as the CAS­(32,34), remain necessary to obtain a suitable reference wave function for subsequent post-CASSCF corrections (PT2 or MRCI) to predict correct spin-state energetics.

Although the Stochastic-MRCI­(96,159) calculations were feasible, more computationally affordable strategies, such as perturbative approaches, remain highly desirable to reduce computational costs, stochastic noise within the FCIQMC algorithm, and memory requirements.

To this end, the CAS­(32,24) space was selected as P , while the total configurational space, T , is identical to that of Stochastic-MRCI­(SD)­(96,159), enabling a direct assessment of the accuracy of Stochastic-SplitGAS. The only difference lies in the use of the SD basis for Stochastic-MRCI­(SD), versus the spin-adapted basis employed in Stochastic-SplitGAS to avoid deviations arising from spin contamination.

Table 7 summarizes the Δ*E* = *E*(^5^A_1g_) – *E*(^3^E_g_) spin gap of the Fe­(II)-porphyrin predicted by different methods. Stochastic-SplitGAS predicts a spin gap of 8.0 kcal/mol, which is only 1.0 kcal/mol larger than the value predicted at the Stochastic-MRCI­(SD) level. The small error with respect to the Stochastic-MRCI­(SD) reference is remarkable, considering the significant diagonal approximation in the QQ block of the SplitGAS Hamiltonian. This result represents a significant improvement over the bare Stochastic-CASSCF­(32,34) prediction of 3.5 kcal/mol, and the qualitatively erroneous predictions arising from CASPT2­(14,16) and Stochastic-CASPT2­(26,27), due to their too small reference active spaces, and suggests that the perturbative SplitGAS approach is able to capture the essential dynamic correlation effects, which are necessary to obtain the correct spin state energetics.

**Table 7:** Spin Gap Δ*E* = *E*(^5^A_1g_) – *E*(^3^E_g_) of Fe­(II)-Porphyrin Predicted by Different Methods

algorithm	Δ*E* [kcal/mol]
ICE-SCF/FR-NEVPT2 ^90^	–7.69^a^
Stochastic-CASSCF(26,27)/CASPT2 ^105^	–2.2
CASSCF(14,16)/CASPT2 ^68^	–0.5
Stochastic-CASSCF(32,34)^68,136^	3.5
Stochastic-CASSCF(40,38)^173^	4.4
Stochastic-CASSCF(32,34) + TDCSD(T)_F12a_ ^174^	5.8
Stochastic-CASSCF(32,34) + MRCI^31^	7.0(1)
P [CAS(32,34)]	8.0(3)

a This calculation employed the geometries reported by Pierloot and colleagues,^196^ whereas all other calculations adopted the geometry reported in Reference 68.

The memory requirements of the PCHB tables within the T space and the SplitGAS Hamiltonian are currently equivalent (see Table 8), as the probabilities are stored in a dense data structure.

**Table 8:** Memory Demand of the PCHB Tables for the T Space and SplitGAS Hamiltonian for the Fe­(II)-Porphyrin Model System^a^

	T		SplitGAS	
data structure	Mem.	Zeros	Mem.	Zeros
dense	97.238 GB	92%	97.238 GB	99%
sparse	10.218 GB	–	1.761 GB	–

a “Dense” and “sparse” refer to implementations using dense (current regime) and sparse (in development) data structures.

Integrals and PCHB-probabilities *p* ^PCHB^ below a threshold of 10^–8^ are truncated or considered zero-values, respectively. However, the amount of zero probabilities strongly increase in the SplitGAS Hamiltonian, readily attributed to the diagonal approximation in the QQ block (eqs 14 and 15). A sparse data structure (see Section 2) would reduce the memory demand for both Stochastic-GAS and Stochastic-SplitGAS, with a more pronounced effect in the latter due to increased sparsity. Consequently, in the sparse-data-structure regime, the applicability of SplitGAS will be extended far beyond GAS, enabling significantly larger T spaces. Moreover, combined with increased Hamiltonian sparsification via orbital localization—and, in the case of GUGA, orbital ordering—this approach could support the correlation of several hundred orbitals, reaching sizes comparable to those accessible by contracted CASPT2, while maintaining the flexibility of uncontracted methods and access to large P reference active spaces.

#### 3.2.3 [Fe­(III)_2_S_2_]^2–^ Model System

Each metal center in the all-ferric [Fe­(III)_2_S_2_]^2–^ system is characterized by a 3d^5^ configuration and a local *s*_loc_ = 5/2 spin. The interaction of the two *s*_loc_ = 5/2 spins leads to six low-lying spin states, with total spin in the *S* ∈ [0, 5] range, of which the *S* ∈ {0, 5} states are collinear and the intermediate *S* ∈ [1, 4] states are noncollinear. In this section, the relative stability of these six energetically low-lying spin states (spin ladder) is investigated at various levels of theory, including Stochastic-SplitGAS.

The main difficulties in studying this and other magnetic multicenter transition metal systems arise from the requirement of a balanced description of the strong electronic correlation across the metal-center 3d orbitals, and the electron delocalization between the metal and the ligand atoms.^197^ Detailed studies have shown that an accurate description of the delocalization is necessary to correctly determine spin coupling constants. It is known that ligand-to-metal charge-transfer (LMCT) interactions have a large impact on the delocalization of the orbitals.^198–200^ The large CAS­(22,26) active space considered in this test case successfully captures strong correlation across the metal centers, as well as electron delocalization between the metal atoms and the bridging sulfur ligands. This active space is readily accessible within the spin-adapted FCIQMC algorithm when Quantum Anamorphosis is applied,^29,168,169^ and is used in the following as the reference for all other approaches that approximate it, including Stochastic-SplitGAS.

Figure 4 illustrates the relative stability of the low-energy states with respect to the singlet ground state (left) for all tested methods, and the deviation from the Stochastic-CASSCF­(22,26) used as reference (right).

**Figure 4:**
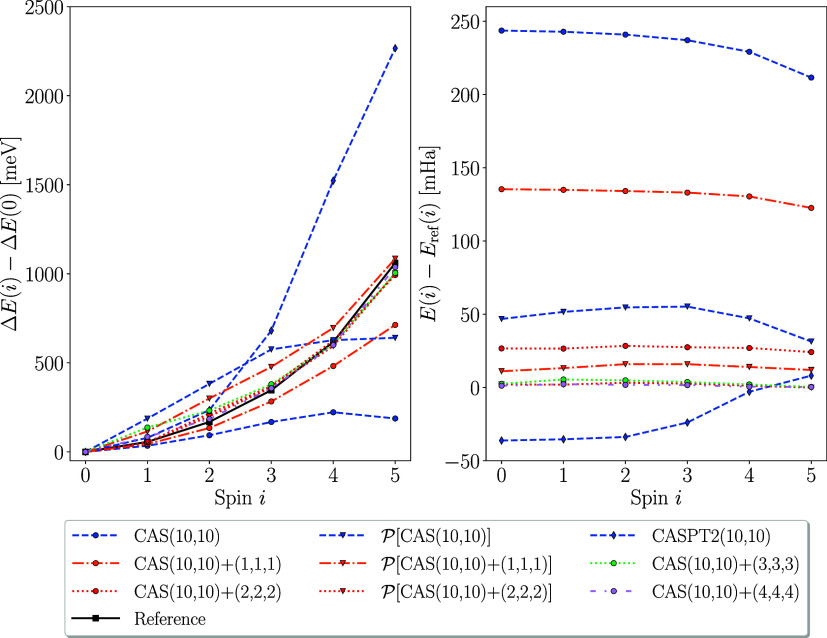
Energies of the energetically lowest spin states of the [Fe­(III)_2_S_2_]^2–^ model system relative to the singlet state (left), and deviations from the CAS­(22,26) reference (right), in a GUGA basis, for the methods utilized in this work. Error bars for the stochastic dynamics are omitted as they are too small compared to the scale of the plot.

The CAS­(10,10) active space represents the smallest space that can qualitatively correctly describe all spin states of the spin ladder. The reduced dimensionality of this reference space allows it to be coupled to a subsequent conventional CASPT2 calculation. In order to rigorously assess the performance of CASPT2­(10,10) with respect to the reference Stochastic-CASSCF­(22,26), we have frozen 82 doubly occupied core orbitals and deleted 32 virtual orbitals both during the CASSCF­(10,10) and in the perturber space of the subsequent CASPT2 calculation. With this setup, CASPT2­(10,10) strictly approximates (and can be directly compared to) the CAS­(22,26) reference, with the perturber space exclusively accounting for excitations from the sulfur bridging atoms, and to the correlating d′ orbitals. The CASSCF­(10,10) reference space does not explicitly account for the metal–ligand electron delocalization nor does it account for the related *superexchange mechanism*,^201,202^ crucial for the correct description of the spin ladder. These limitations lead to a spin ladder that substantially deviates from the Heisenberg quadratic trend (see Figure 4). In contrast, superexchange and metal–ligand delocalization effects are perturbatively accounted for by CASPT2, which regains the quadratic Heisenberg trend. However, the comparison with the CAS­(22,26) reference reveals that CASPT2­(10,10) significantly overstabilizes the low-spin states, leading to a too steep spin ladder. The CASPT2 overstabilization of the low-spin states in polynuclear transition metal clusters has already been documented in the literature.^203^ It has also been observed by one of us in the context of the quantum chemical prediction of the spin ladder of a trinuclear [Mn­(IV)_3_O_4_]^4+^ model system.^170^ Le Guennic and co-workers have studied the role of the IPEA shift in CASPT2,^203^ and have suggested to utilize larger IPEA values to reduce the overstabilization. Figure 5 of reference 170 indeed shows that the overstabilization of the low-spin states is reduced by increasing the IPEA shift. It is worth noting that the overstabilization of the low-spin states is characteristic of polynuclear transition metal clusters, including the [Fe(III)_2_S_2_]^2–^ dimer, while the opposite trend (overstabilization of high-spin states) is observed for single-center transition-metal complexes, as shown in the present work for the Fe­(II)-porphyrin test case, predicted by the smaller CASSCF­(14,16) calculations. In contrast to CASPT2­(10,10), the SplitGAS perturbative strategy (labeled as P ­[CAS­(10,10)] in Figure 4), where the P space consists of the CAS­(10,10) wave function, does not exhibit any overstabilization of the low-spin states. However, it retains the unphysical shape of the spin ladder predicted by the CASSCF­(10,10) space.

It is possible to improve over the CASPT2­(10,10) and the SplitGAS with P ­[(10,10)] by extending the P space. It is known that the inclusion of LMCT states into the active space is crucial as they give rise to notable metal–ligand delocalization.^197–199,204,205^ Malrieu, Calzado, Cabrero and co-workers, showed that the delocalization is linked to the 1*h* and 2*h* + 1*p* excitations in DDCI language, where 1*h* excitations correspond to LMCT states. Inclusion of 2*h* + 1*p* excitations significantly increases the weights of the LMCT states via high-order interactions.^198^ The works of Broer and co-workers^199,200,206^ as well as Giner and co-workers^197,205^ have linked this to orbital relaxation of the LMCT states. Also relevant is the work of Arantes and Taylor where the CI expansion within a set of neutral configurations is enhanced by the addition of configurations that results from single excitations out of the neutral set.^207^ Both 1*h* and 2*h* + 1*p* configurations are included in the Q space corresponding to all double excitations on top of CAS­(10,10) but no interaction occurs between them due to the diagonal approximation in QQ within SplitGAS. We can leverage the flexibility of the GAS algorithm and extend the definition of the P space to include single excitations from the GAS3 space (orbitals of the sulfur bridging atoms) and into the GAS2 space (correlating d’ orbitals on the metal centers), labeled as CAS­(10,10)+(1,1,1) in Figure 4. The CAS­(10,10)+(1,1,1) space is equivalent to a DDCI1 space^208^ with a CAS­(10,10) minimal space and outer 1*h*, 1*p* and 1*h* + 1*p* excitations. Outer excitations of 2*h* + 1*p* type are again not explicitly included into the P space, but due to the inclusion of 1*h* configurations in P an interaction is established via the connecting elements in the PQ and QP blocks. Hence, a perturbative inclusion of the relaxation effects due to the 2*h* + 1*p* configurations can be achieved, given the uncontracted nature of SplitGAS. A similarly truncated wave function can be constructed within the RAS framework. Due to the contracted nature of CASPT2/RASPT2, a RASPT2 within the (22,26) reference space is not possible. The RAS zeroth-order wave function already spans the available orbital space and cannot account for additional contributions as there are no further orbitals beyond the CAS­(22,26). The RASPT2 is thus omitted from Figure 4. While the CAS­(10,10) + (1,1,1) total energies are substantially far from the CAS­(22,26) reference, the relative energetics are significantly improved, with the restoration of the Heisenberg quadratic trend. It is in much better agreement with the CAS­(22,26) reference, both as compared to CASSCF­(10,10), CASPT2­(10,10) and SplitGAS with P ­[CAS­(10,10)]. Further substantial improvement, both in terms of total and relative energies, is provided by SplitGAS with P ­[CAS­(10,10) + (1,1,1)]. Considering that in SplitGAS with P ­[CAS­(10,10) + (1,1,1)] the Q space contains configurations with up to triple particle excitations from the CAS­(10,10) space, it can be considered as a perturbative approximation of the CAS­(10,10) + (3,3,3) wave function, also reported in Figure 4. This space leads to total energies and relative energetics that are nearly indistinguishable from the CAS­(22,26) reference, highlighting the amount of correlation effectively missing in the SplitGAS with P ­[CAS­(10,10) + (1,1,1)] wave function. If we expand the P space even further and include up to double excitations between the GAS subspaces, the SplitGAS results becomes practically indistinguishable from both T = CAS­(10,10) + (4,4,4) and the CAS­(22,26) reference.

The improvement in the SplitGAS energy predictions is also reflected in the wave function coefficients. Figure 5 illustrates the instantaneous CI coefficients (after reaching stationary conditions) in the P space for the CAS­(10,10) + (2,2,2) and CAS­(10,10) + (3,3,3) wave functions of the singlet spin state of [Fe­(III)_2_S_2_]^2–^, as well as the deviations obtained when solving the P spaces alone ([CAS­(10,10)] and [CAS­(10,10) + (1,1,1)], respectively) and the corresponding SplitGAS wave functions.

**Figure 5:**
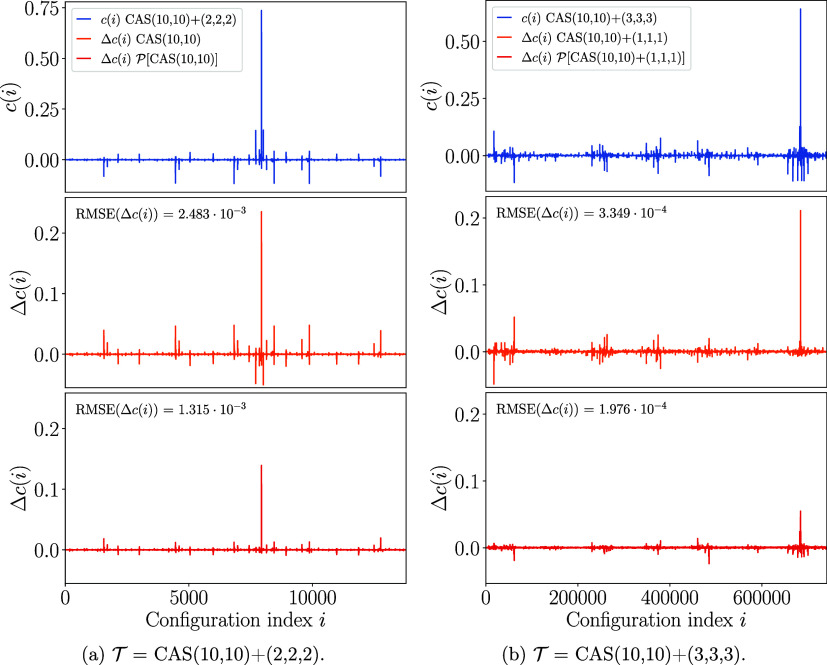
Coefficients in the respective P spaces of the CAS­(10,10) + (2,2,2) and CAS­(10,10) + (3,3,3) spin-adapted wave functions, and deviations obtained when solving only the P space or via SplitGAS.

For both configurational spaces, CAS­(10,10) + (2,2,2) and CAS­(10,10) + (3,3,3), the SplitGAS wave function recovers a substantial portion of the total wave function, as indicated by the reduced deviations from the total space, and by the nearly halving of the root-mean-square error (RMSE) in SplitGAS as compared to the optimization of the P space alone. The remaining deviations in SplitGAS stem from two main sources: the inherent perturbative approximation of SplitGAS and the stochastic noise of the FCIQMC algorithm, which affects both the full and SplitGAS dynamics. These contributions can be largely decoupled by time-averaging the wave functions (see Section SI.S2 for details).

It is worth comparing the stochastic noise that characterizes the Stochastic-SplitGAS dynamics and the dynamics over the full T space with no diagonal approximation. Figure 6 shows the imaginary-time evolution, upon stationary conditions have been reached, for the lowest singlet spin state, using a total of 10^5^ walkers and 1000 configurations in the semistochastic space, for the two dynamics.

**Figure 6:**
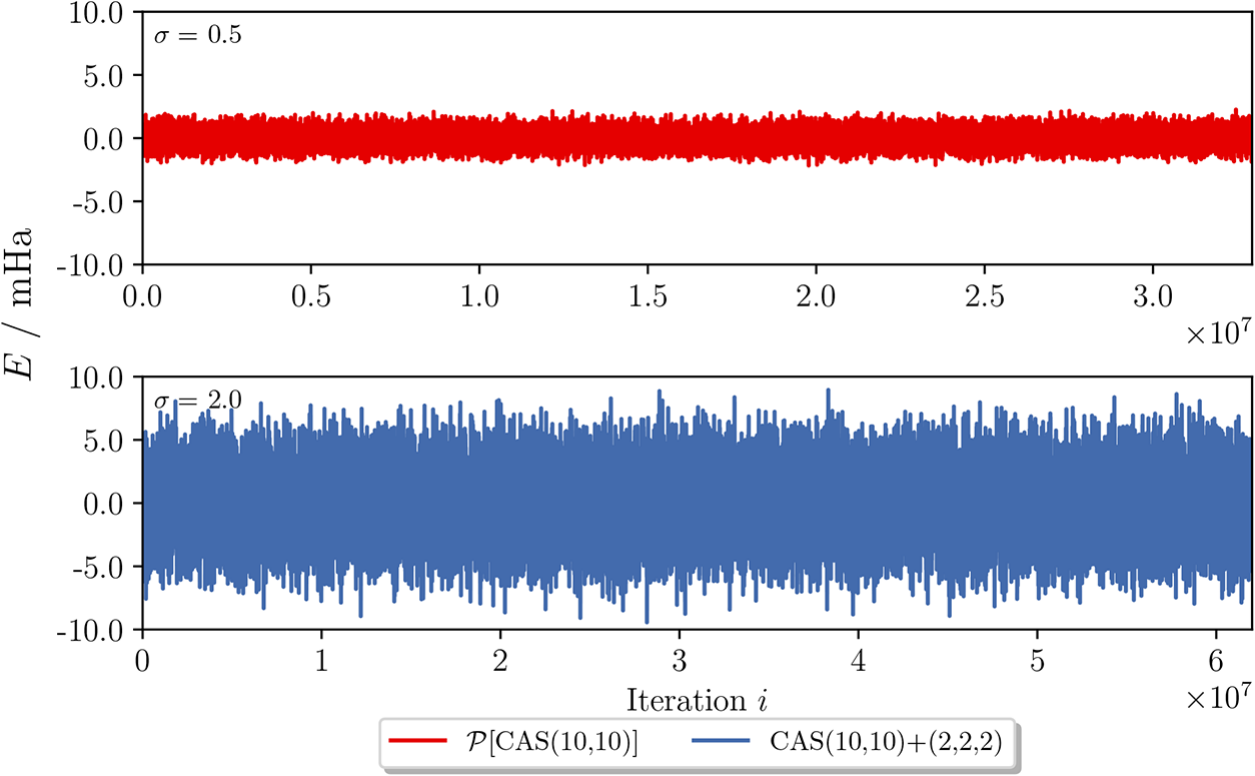
FCIQMC dynamic of the Stochastic-SplitGAS, and corresponding full T space for the singlet spin state of the [Fe­(III)_2_S_2_]^2–^ model system, using a GUGA basis, centered around their mean value (GAS = −5092.921 Ha, SplitGAS = −5092.905 Ha).

The SplitGAS dynamics shows reduced stochastic noise, as compared to the T dynamics. This is particularly interesting as the SplitGAS dynamics evolves with a time-step Δτ = 5.879 · 10^–4^ i · ℏ/(Ha · s) that is nearly double the one for the T dynamics (Δτ = 3.568 · 10^–4^ i · ℏ/(Ha · s)). With all parameters being equal for the two dynamics, a larger Δ*τ* would usually increase stochastic noise; yet, SplitGAS shows the opposite trend while sampling the same configurational space. Greater rigor is achieved by comparing the efficiency, η, of both calculations, obtained via the variance reduction per wall-clock time *t*

27 $$\eta ={\left(\mathrm{Var}\left(E_{\mathrm{proj}}\right)\cdot t\right)}^{-1}$$

GAS and SplitGAS calculations were performed both in CSF and SD basis after reaching stationary condition over a period of 5600 min. The ratios between the efficiency of the SplitGAS and GAS calculations in the GUGA and SD basis are 13.4 ± 0.8 and 8.4 ± 0.6, respectively, indicating a remarkable increase in efficiency when using SplitGAS.

The spin ladder of [Fe­(III)_2_S_2_]^2–^ exhibits significant spin-contamination in the SD basis (see Figure 7). However, the impact on the spin-gap between the collinear *S* ∈ {0, 5} states is negligible (see Appendix [Sec app1-sec2] for details).

**Figure 7:**
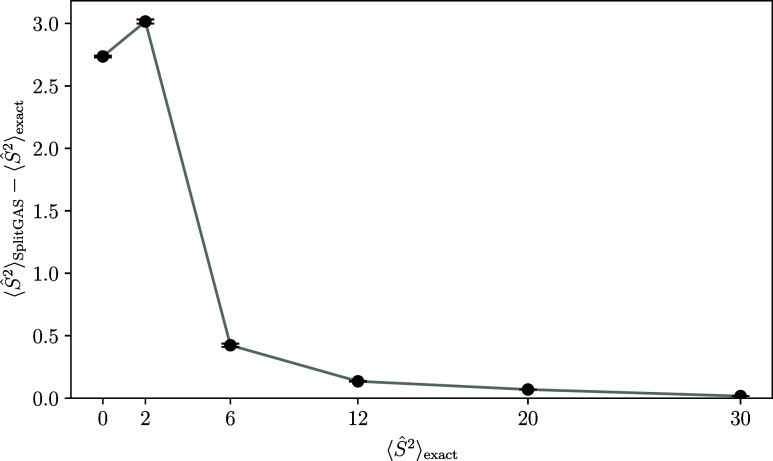
Deviation of the ⟨*Ŝ* ^2^⟩ expectation value obtained via SplitGAS ( P ­[(10,10)+(1,1,1)]) from the spin-pure solution for [Fe­(III)_2_S_2_]^2–^.

## 4 Conclusions

In this work, a stochastic extension of the perturbative SplitGAS approach has been presented, named Stochastic-SplitGAS. The method relies on the GAS concept for the partitioning of the configuration interaction space into principal, P , and perturber, Q , spaces, and on Löwdin downfolding, truncated to the second order for the construction of an effective Hamiltonian, approximated in the QQ space.

The Stochastic-SplitGAS algorithm relies and builds on the Stochastic-GAS algorithm; in that, the excitation generation probabilities are generated and stored in PCHB tables for each supergroup and probabilities violating either the GAS or the SplitGAS constraints are set to zero. The Stochastic-GAS flexibility in the construction of the P and Q spaces is transferred to Stochastic-SplitGAS, with allowed supergroups chosen on the basis of local or cumulative constraints.

In Stochastic-SplitGAS, no additional information is stored in memory and no additional operations are required as compared to Stochastic-GAS; thus, the two methods are currently similar both in terms of memory requirements and runtime. A sparse data structure of the PCHB tables is possible, and once implemented it will significantly reduce memory requirements of both Stochastic-GAS and Stochastic-SplitGAS, differentially favoring the latter strategy. While the wall clock time per iteration of Stochastic-GAS and Stochastic-SplitGAS is similar, the diagonal approximation in the QQ block considerably reduces stochastic noise and dramatically enhances the efficiency of the latter, as demonstrated for the [Fe­(III)_2_S_2_]^2–^ system.

The ^5^A_1g_–^3^E_g_ spin gap in the Fe­(II)-porphyrin system, and the spin ladder in the [Fe­(III)_2_S_2_]^2–^ cluster numerically demonstrate the applicability of the method in capturing dynamic correlation effects in large active spaces, and predicting the correct spin state ordering. For both systems, perturbed energetics and wave functions are in close agreement with those obtained by the corresponding Stochastic-GAS, where no diagonal approximation in the QQ block is made, and are largely improved as compared to the optimization of the P space alone. The reduction in the RMSE in approximating the CI coefficients of the T space for the ozone and of the P space of the [Fe­(III)_2_S_2_]^2–^ systems are particularly striking.

The test cases presented in this work demonstrate a 2-fold superiority of Stochastic-SplitGAS over CASPT2. First, the method does not suffer from any artificial overstabilization of the low spin states for poly nuclear transition metal clusters, which has been reported for CASPT2 both for the [Fe(III)_2_S_2_]^2–^ (this work) and for the trinuclear [Mn(IV)_3_O_4_]^4−^ cluster. Second, in contrast to CASPT2, Stochastic-SplitGAS can be coupled to large P reference wave functions, owing to the fact that the method does not require high-order reduced density matrices; striking is the direct comparison with our earlier contracted Stochastic-CASPT2. Stochastic-CASPT2 with time-averaged reference wave functions could only handle up to CAS­(26,27) zeroth-order wave functions, which is not large enough to qualitatively reproduce the ^5^A_1g_–^3^E_g_ spin gap in the Fe­(II)-porphyrin model system. On the contrary, Stochastic-SplitGAS could retain the CAS­(32,34) reference wave functions, which was shown to be necessary for a qualitatively correct description of this system. A ^5^A_1g_–^3^E_g_ energy gap of 8.0 kcal/mol was obtained with Stochastic-SplitGAS, which is in excellent agreement with the 7.0 kcal/mol gap obtained by the more demanding (see efficiency analysis) Stochastic-MRCI­(SD) performed on the same configurational space. The Fe­(II)-porphyrin test case, with a total of 96 electrons perturbatively correlated in a space of 159 orbitals demonstrates the scalability of the method.

Despite the absence of a sparse data structure for the PCHB tables, the results presented in this work are already very promising, both in terms of accuracy of the method and in terms of computational efficiency. The results obtained motivate us to pursue further algorithmic improvements, especially the implementation of a sparse representation of the PCHB excitation probabilities, leading to substantially reduced memory requirements and larger principal and perturber spaces. A straightforward application to even larger PNTM clusters, such as the FeMo-cofactor and P-cluster of nitrogenases, as well as the Mn_12_ single-molecule magnet, is envisioned.

## Supplementary Material





## Appendix

Below, operators are expressed as matrices in a finite basis to facilitate the concepts of partitioning and “blocks”. The matrix representation of an operator *Ô* is written in bold characters, i.e., **O**.

### A.1 Diagonal Approximation

In the following, we will demonstrate that the proposed effective Hamiltonian is equivalent to the second order truncated Löwdin equation (eq 16). Consider the following eigenvalue equation from variational theory partitioned into P and Q contributions

28
$$\left(\begin{array}{ll} H^{\mathrm{PP}} & H^{\mathrm{PQ}}\\ H^{\mathrm{QP}} & H^{\mathrm{QQ}} \end{array}\right)\left(\begin{array}{l} c^{P}\\ c^{Q} \end{array}\right)=E\left(\begin{array}{ll} S^{\mathrm{PP}} & S^{\mathrm{PQ}}\\ S^{\mathrm{QP}} & S^{\mathrm{QQ}} \end{array}\right)\left(\begin{array}{l} c^{P}\\ c^{Q} \end{array}\right)$$

We assume an orthonormal basis (**S** = **I**) which leads to

29
$$H^{\mathrm{PP}}c^{P}+H^{\mathrm{PQ}}c^{Q}=EI^{\mathrm{PP}}c^{P}$$

30
$$H^{\mathrm{QP}}c^{P}+H^{\mathrm{QQ}}c^{Q}=EI^{\mathrm{QQ}}c^{Q}$$

Let us solve for $c^{Q}$

31
$$c^{Q}={\left(EI^{\mathrm{QQ}}-H^{\mathrm{QQ}}\right)}^{-1}H^{\mathrm{QP}}c^{P}$$

We approximate **H** ^*QQ*^ to be a diagonal matrix ${\mathrm{H̅}}^{\mathrm{QQ}}$ (Figure 2)­

32
$${\mathrm{H̅}}^{\mathrm{QQ}}=\mathrm{diag}\left(H^{\mathrm{QQ}}\right)$$

Thus, the inversion (*E* **I** ^QQ^ – **H̅** ^QQ^)^−1^ becomes trivial. Insertion of eq 30 into eq 28 yields an effective Hamiltonian of dimensionality dim (P)

33
$$\underset{\mathrm{H̃}}{\underset{︸}{(H^{\mathrm{PP}}+H^{\mathrm{PQ}}\left(EI^{\mathrm{QQ}}-{\mathrm{H̅}}^{\mathrm{QQ}}{)}^{-1}H^{\mathrm{QP}}\right)}}c^{P}=EI^{\mathrm{PP}}c^{P}$$

The individual elements of **H̃** are obtained as

34
H̃ij=HijPP+∑α∈QHiαPQ∑β∈Q(E−HQQ)αβ−1HβjQPδαβ=HijPP+∑α∈QHiαPQ(E−HQQ)αα−1HαjQP=HijPP+∑α∈QHiαPQHαjQPE−HααQQ

Direct comparison of eq 33 with eq 16 shows that our initial statement holds true.

### Definitions

We partition the full Hamiltonian **H** and **S** ^**2**^ into four blocks

35
$$H=\left(\begin{array}{ll} H^{\mathrm{PP}} & H^{\mathrm{PQ}}\\ H^{\mathrm{QP}} & H^{\mathrm{QQ}} \end{array}\right),,S^{2}=\left(\begin{array}{cc} {\left(S^{2}\right)}^{\mathrm{PP}} & 0^{\mathrm{PQ}}\\ 0^{\mathrm{QP}} & {\left(S^{2}\right)}^{\mathrm{QQ}} \end{array}\right)$$

The **S** ^**2**^ matrix is block-diagonal in P and Q since the subspaces are separated by particle transfer, and *Ŝ* ^2^ only couples SDs via exchange (α, β ↔ β, α), i.e., orbital occupations are preserved. Since the full Hamiltonian **H** commutes with **S** ^**2**^

36
$$\left[H,S^{2}\right]=0$$

the two operator admit common eigenstates. The goal here is to show that in general [**H̃**, **S** ^**2**^] ≠ 0, where **H̃** is the effective SplitGAS Hamiltonian.

### Procedure

Given the block-diagonal structure of **S** ^**2**^ we have to consider the commutators,

37
$$\left[H,S^{2}\right]=\left(\begin{array}{ll} {}[H,S^{2}{]}_{\mathrm{PP}} & [H,S^{2}{]}_{\mathrm{PQ}}\\ {}[H,S^{2}{]}_{\mathrm{QP}} & [H,S^{2}{]}_{\mathrm{QQ}} \end{array}\right)$$

Note that we use a slightly generalized notation for the commutators here, as both $H^{\mathrm{PQ}}$ and $H^{\mathrm{QP}}$ are rectangular. We define

$$[H,S^{2}{]}_{\mathrm{xy}}=H^{\mathrm{xy}}{\left(S^{2}\right)}^{\mathrm{yy}}-{\left(S^{2}\right)}^{\mathrm{xx}}H^{\mathrm{xy}}=\{\begin{array}{lr} \left[H^{\mathrm{xx}},{\left(S^{2}\right)}^{\mathrm{xx}}\right], & \;x=y\\ H^{\mathrm{xy}}{\left(S^{2}\right)}^{\mathrm{yy}}-{\left(S^{2}\right)}^{\mathrm{xx}}H^{\mathrm{xy}},\; & x\neq y \end{array}$$
38

We know that all these commutators are zero for the full Hamiltonian because of eq 35. In the effective SplitGAS Hamiltonian the block $H^{\mathrm{QQ}}$ is approximated by a diagonal matrix (eq 31). We only have to consider the commutator $\left[{\mathrm{H̅}}^{\mathrm{QQ}},{\left(S^{2}\right)}^{\mathrm{QQ}}\right]$ as all other commutators remain unchanged compared to the original matrix. Under the diagonal approximation, the Slater determinants become eigenvectors within the ${\mathrm{H̅}}^{\mathrm{QQ}}$ block, and its eigenvalues are given by the diagonal matrix elements

39
$$\varepsilon_{\alpha }=\left\langle q_{\alpha }\right|H\left|q_{\alpha }\right\rangle$$

If |*q*_α_⟩ and |*q*_β_⟩ are coupled through *Ŝ* ^2^, but *ε*_*α*_ ≠ *ε*_*β*_, then they are eigenvectors of ${\mathrm{H̅}}^{\mathrm{QQ}}$ but not of ${\left(S^{2}\right)}^{\mathrm{QQ}}$ , implying that the commutator is non-zero, $\left[{\mathrm{H̅}}^{\mathrm{QQ}},{\left(S^{2}\right)}^{\mathrm{QQ}}\right]\neq 0$ .

The only exception to this is the rare event where all eigenvalues ε_α_ are degenerate and hence ${\mathrm{H̅}}^{\mathrm{QQ}}=\varepsilon_{\alpha }I^{\mathrm{QQ}}$ . In such a case, the eigenvalues of ${\mathrm{H̅}}^{\mathrm{QQ}}$ are rotationally invariant and a common set of eigenvectors with ${\left(S^{2}\right)}^{\mathrm{QQ}}$ exists. A numerical analysis of the ⟨*Ŝ* ^2^⟩ expectation values of ozone and [Fe­(III)_2_S_2_]^2–^ was performed to confirm and investigate the aforementioned analysis. The resulting expectation values for ozone are listed in Table 9.

**Table 9:** ⟨*Ŝ* ^2^⟩ Expectation Value of Ozone for Different P Spaces

	⟨*Ŝ* ^2^⟩_SplitGAS_	
⟨*Ŝ* ^2^⟩_exact_	P [CAS(4,3)]	P [CAS(4,3) + (1,1,2)]
0	0.172	0.055
2	2.094	2.033

We observe significant spin contamination in the P[CAS(4,3)] space, which is reduced (but not eliminated) in the larger P ­[CAS­(4,3) + (1,1,2)] space.

We performed a similar analysis for the [Fe­(III)_2_S_2_]^2–^ system where spin is of pivotal importance. The deviations of the ⟨*Ŝ* ^2^⟩ expectation values from the spin-pure solutions are shown in Figure 7.

A significant spin-contamination is observed, which, as expected, reduces for the higher spin states (in line with the results reported in Table 9 for the ozone). Remarkably, the singlet-undecet energy gap of 39 mHa doesn’t significantly deviate from the GUGA value of 40 mHa, with the latter being spin pure.

## Notes

(a) The geometry of ozone in Cartesian coordinates, (b) representative input files for the quintet/triplet SplitGAS calculations of Fe­(II)-porphyrin and the GAS/SplitGAS calculations of the [Fe­(III)_2_S_2_]^2–^ model system, (c) the total energies for all GUGA calculations, and (d) an analysis of the histogrammed and total wave function of [Fe­(III)_2_S_2_]^2–^ (PDF)
Archive is provided, which contains all orbital and integral files for ozone and [Fe­(III)_2_S_2_]^2–^ (ZIP)
