# Supplementary material for: Stochastic-SplitGAS: A Quantum Monte Carlo Multi-Reference Perturbation Theory Based on the Imaginary-Time Evolution of Effective Hamiltonians
Source: J Chem Theory Comput. 2025 Dec 3;21(24):12523–44. doi: 10.1021/acs.jctc.5c01270 (PMC12746464; doi:10.1021/acs.jctc.5c01270)
Supplement: Supplementary file 1 [file ct5c01270_si_001.pdf]

Stochastic-SplitGAS: A Quantum Monte Carlo Multi-Reference Perturbation Theory Based on the Imaginary-Time Evolution of Effective Hamiltonians (Supporting Information)

Luca Bonfirraro,[*,†] Oskar Weser,[†,‡] Maru Song,[†] and Giovanni Li Manni[*,†]

†*Max Planck Institute for Solid State Research, Heisenbergstr. 1, 70569 Stuttgart, Germany*

‡*Massachusetts Institute of Technology, 77 Massachusetts Ave, Cambridge, MA 02139, USA*

E-mail: l.bonfirraro@fkf.mpg.de; g.limanni@fkf.mpg.de

S1 Computational Details

The following inputs have been tested using commit `d195681e6` of the NECI software package, keywords for other versions may vary.

S1.1 Ozone

The cartesian coordinates of Ozone are given below:

Ozone coordinates

```
3
O 0.0 0.0 0.0
O 0.0 -1.0837773359460974 0.6673415195307271
O 0.0 1.0837773359460974 0.6673415195307271
```

The localized and reordered orbitals used in the CI calculation as well as the integrals can be found in `ozone.LocOrb` and `ozone.FciDmp` respectively.

S1.2 Fe(II)-porphyrin

The Stochastic-SplitGAS inputs for both the triplet and singlet using NECI are given below.

quintet.input

```
Title

System read
    electrons 96
    nonuniformrandexcits guga-gas-pchb manual \
    drawing unif:unif unif-unif:fast-fast \
    weighting uniform initial
    nobrillouintheorem
    guga 4
    FCIDUMP-name quint.FciDmp
```

```
freeformat

GAS-SPEC CUMULATIVE 3
    32 62 64
    34 94 96
    93 96 96
    9*1  6*2  21*3 \
    8*1  2*2  17*3 \
    8*1  2*2  17*3 \
    6*1  3*2  13*3 \
      1  6*2   9*3 \
         6*2   6*3 \
         6*2   6*3 \
         3*2   4*3

GAS-PQ-TRUNCATION CUMULATIVE 3
    32 64 64
    34 96 96
    93 96 96
    9*1  6*2  21*3 \
    8*1  2*2  17*3 \
    8*1  2*2  17*3 \
    6*1  3*2  13*3 \
      1  6*2   9*3 \
         6*2   6*3 \
```

```
                6*2    6*3  \
                3*2    4*3
endsys

calc
    definedet  \
            1-18  19-20  21  23-24  \
           73-88  89-90  \
         127-142  143-144  \
         181-192  193-194  195  \
         225-226  227-228  229-230  231-232  \
                  257-258  259-260  261  \
                  281  283-284  285-286  \
                  305-306  307-308

    totalwalkers  1e8
    semi-stochastic  1
    read-core
    readpops
    walkcontgrow

    tau-values   start  from-popsfile
    tau-search   algorithm  histogramming  \
                 stop-condition  off  \
                 maxwalkerbloom  1
```

```
    methods
        method vertex fcimc
    endmethods

    auto-adaptive-shift
    aas-matele2
    as-offset -1951.5

    truncate-spawns 3
    diagshift .00
    shiftdamp .02
    nmcyc 200000
    proje-changeref 1.6
    truncinitiator
    addtoinitiator  3
    allrealcoeff
    realspawncutoff .30
    memoryfacspawn 10.00
    memoryfacpart 5.00
    time 2800

endcalc

logging
```

```
    highlypopwrite 50
    hdf5-pops
endlog
end
```

triplet.input

```
Title

System read
    electrons  96
    nonuniformrandexcits guga-gas-pchb manual \
    drawing unif:unif unif-unif:fast-fast \
    weighting uniform initial
    nobrillouintheorem
    guga 2
    FCIDUMP-name tripl.FciDmp
    freeformat

    GAS-SPEC CUMULATIVE 3
        32 62 64
        34 94 96
        93 96 96
        9*1  6*2  21*3 \
        8*1  2*2  17*3 \
```

```
        8*1   2*2   17*3 \
        6*1   3*2   13*3 \
          1   6*2    9*3 \
              6*2    6*3 \
              6*2    6*3 \
              3*2    4*3

    GAS-PQ-TRUNCATION CUMULATIVE 3
        32 64 64
        34 96 96
        93 96 96
        9*1   6*2   21*3 \
        8*1   2*2   17*3 \
        8*1   2*2   17*3 \
        6*1   3*2   13*3 \
          1   6*2    9*3 \
              6*2    6*3 \
              6*2    6*3 \
              3*2    4*3

endsys

calc
    definedet \
           1-18 19-20 21 23-24 \
```

```
          73-88 89-90 \
        127-142 143-144 \
        181-192 193-194 195 \
        225-226 227-228 229-230 231-232 \
                257-258 259-260 261 \
                281 283-284 285-286 \
                305-306 307-308

totalwalkers 1e8
semi-stochastic 1
read-core
readpops
walkcontgrow

tau-values   start from-popsfile
tau-search   algorithm histogramming \
             stop-condition off \
             maxwalkerbloom 1

methods
    method vertex fcimc
endmethods

auto-adaptive-shift
aas-matele2
```

```
    as-offset -1951.35

    truncate-spawns 3
    diagshift .00
    shiftdamp .02
    nmcyc 200000
    proje-changeref 1.6
    truncinitiator
    addtoinitiator  3
    allrealcoeff
    realspawncutoff .30
    memoryfacspawn 10.00
    memoryfacpart 5.00
    time 2800

endcalc

logging
    highlypopwrite 50
    hdf5-pops
endlog
end
```

S1.3 $[Fe(III)_2S_2]^{2-}$

The orbitals and integrals of the respective $[Fe(III)_2S_2]^{2-}$ calculations are found in `fe2s2_22in26_s{spin}.LocOrb_reorder` and `fe2s2_22in26_s{spin}.FciDmp`

Exemplary inputs for a CAS(10,10)CI and Stochastic-SplitGAS calculation with a CAS(10,10) $\mathcal{P}$ space are given below.

CAS(10,10)

```
Title

System read
    electrons 22
    nonuniformrandexcits guga-gas-pchb localised
    nobrillouintheorem
    guga 0
    FCIDUMP-name fe2s2_22in26_s1.FciDmp
    freeformat

    GAS-SPEC LOCAL 3
        10 10 10
        10 0 0
        6  12 12
        5*1 5*2 5*1 5*2 6*3

endsys
```

```
calc
    definedet 1 3 5 7 9 22 24 26 28 30 41-52

    totalwalkers 1e6
    semi-stochastic 20000
    pops-core 1000
    readpops
    walkcontgrow

    tau-values  start from-popsfile
    tau-search  algorithm histogramming \
                stop-condition off \
                maxwalkerbloom 2

    methods
        method vertex fcimc
    endmethods

    truncate-spawns 5
    diagshift .00
    shiftdamp .02
    nmcyc 60000
    proje-changeref 1.6
    truncinitiator
    addtoinitiator  3
```

```
    allrealcoeff
    realspawncutoff .30
    memoryfacspawn 200.00
    memoryfacpart 100.00
    time 110

endcalc

logging
    highlypopwrite 50
    hdf5-pops
endlog
end
```

$\mathcal{P}$ [CAS(10,10)]

```
Title

System read
    electrons  22
    nonuniformrandexcits guga-gas-pchb localised
    nobrillouintheorem
    guga 0
    FCIDUMP-name fe2s2_22in26_s1.FciDmp
```

```
    freeformat

    GAS-SPEC LOCAL 3
        10  0  20
        10  0  20
        6   0  12
        5*1  5*2  5*1  5*2  6*3

    SplitGAS-SPEC LOCAL 3
        10  9  11
        10  0  1
        6   11  12
        5*1  5*2  5*1  5*2  6*3

endsys

calc
    definedet  1  3  5  7  9  22  24  26  28  30  41-52

    totalwalkers  1e6
    semi-stochastic  50000
    pops-core  1000
    readpops

    tau-values   start  from-popsfile
```

```
    tau-search  algorithm histogramming \
                stop-condition off \
                maxwalkerbloom 2

    methods
        method vertex fcimc
    endmethods

    truncate-spawns 5
    shiftdamp .02
    nmcyc 150000
    proje-changeref 1.6
    truncinitiator
    addtoinitiator  3
    allrealcoeff
    realspawncutoff .30
    memoryfacspawn 10.00
    memoryfacpart 5.00
    time 2800

endcalc

logging
    highlypopwrite 50
    hdf5-pops
```

```
endlog
end
```

The walker numbers used to converge the respective calculations are given in Table S1.

Table S1: Walker numbers used to converge the $[Fe(III)_2S_2]^{2-}$ calculations.

CAS(10,10)	CAS(10,10)+(1,1,1)	CAS(10,10)+(2,2,2)
$1 \cdot 10^5$	$2 \cdot 10^6$	$5 \cdot 10^6$
CAS(10,10)+(3,3,3)	CAS(10,10)+(4,4,4)	$\mathcal{P}$ [CAS(10,10)]
$1 \cdot 10^7$	$5 \cdot 10^7$	$1 \cdot 10^6$
$\mathcal{P}$ [CAS(10,10)+(1,1,1)]	$\mathcal{P}$ [CAS(10,10)+(2,2,2)]	CAS(10,10)+CASPT2
$1 \cdot 10^7$	$5 \cdot 10^7$	-

S2 Results

S2.1 Ozone

The total energies obtained for ozone are listed in Table S2.

Table S2: Total energies obtained for ozone in Hartree.

CAS(4,3)+(1,1,2)	$\mathcal{P}$[CAS(4,3)+(1,1,2)]	CAS(12,9)
-224.6625	-224.7200	-224.7252

S2.2 Fe(II)-porphyrin

The total energies obtained for ozone are listed in Table S2.

Table S3: Total energies obtained for Fe(II)-porphyrin in Hartree.

triplet	quintet
-1951.9374	-1951.9248

S2.3 $[Fe(III)_2S_2]^{2-}$

The total energies of the $[Fe(III)_2S_2]^{2-}$ spin-ladder are listed in Table S4.

Table S4: Total energies of the $[Fe(III)_2S_2]^{2-}$ spin-ladder in Hartree

Spin i	CAS(10,10)	CAS(10,10)+(1,1,1)	CAS(10,10)+(2,2,2)
0	-5092.7082	-5092.8165	-5092.9252
1	-5092.7070	-5092.8149	-5092.9232
2	-5092.7048	-5092.8116	-5092.9173
3	-5092.7021	-5092.8061	-5092.9116
4	-5092.7001	-5092.7988	-5092.9022
5	-5092.7013	-5092.7903	-5092.8887
	CAS(10,10)+(3,3,3)	CAS(10,10)+(4,4,4)	$\mathcal{P}$[CAS(10,10)]
0	-5092.9494	-5092.9507	-5092.9050
1	-5092.9443	-5092.9475	-5092.8982
2	-5092.9408	-5092.9439	-5092.8910
3	-5092.9354	-5092.9375	-5092.8839
4	-5092.9271	-5092.9286	-5092.8820
5	-5092.9124	-5092.9125	-5092.8815
	$\mathcal{P}$[CAS(10,10)+(1,1,1)]	$\mathcal{P}$[CAS(10,10)+(2,2,2)]	CAS(10,10)+CASPT2
0	-5092.9408	-5092.9498	-5092.9881
1	-5092.9366	-5092.9478	-5092.9852
2	-5092.9297	-5092.9424	-5092.9794
3	-5092.9232	-5092.9365	-5092.9630
4	-5092.9152	-5092.9279	-5092.9321
5	-5092.9009	-5092.9129	-5092.9048

An analysis of the time averaged coefficients in $\mathcal{P}$ can be found below.

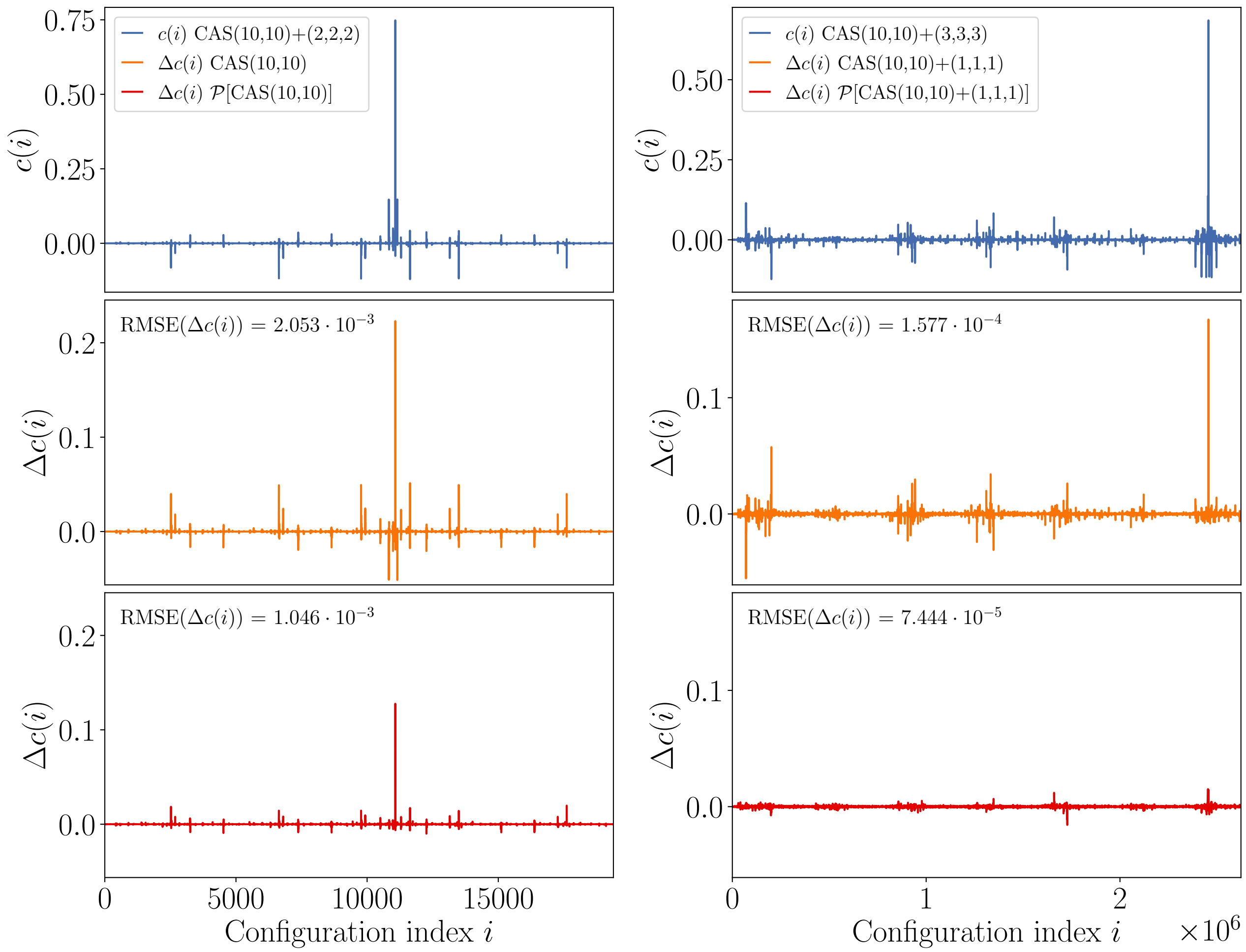

(a) $\mathcal{T}$ =CAS(10,10)+(2,2,2)

(b) $\mathcal{T}$ = CAS(10,10)+(3,3,3)

Figure S1: Time averaged wave function coefficients of two total spaces and the deviation of the respective $\mathcal{P}$ and SplitGAS wave functions from the total wave function.

An analysis of the CI coefficients in the total $\mathcal{T}$ space instead of $\mathcal{P}$ can be found in Fig. S2.

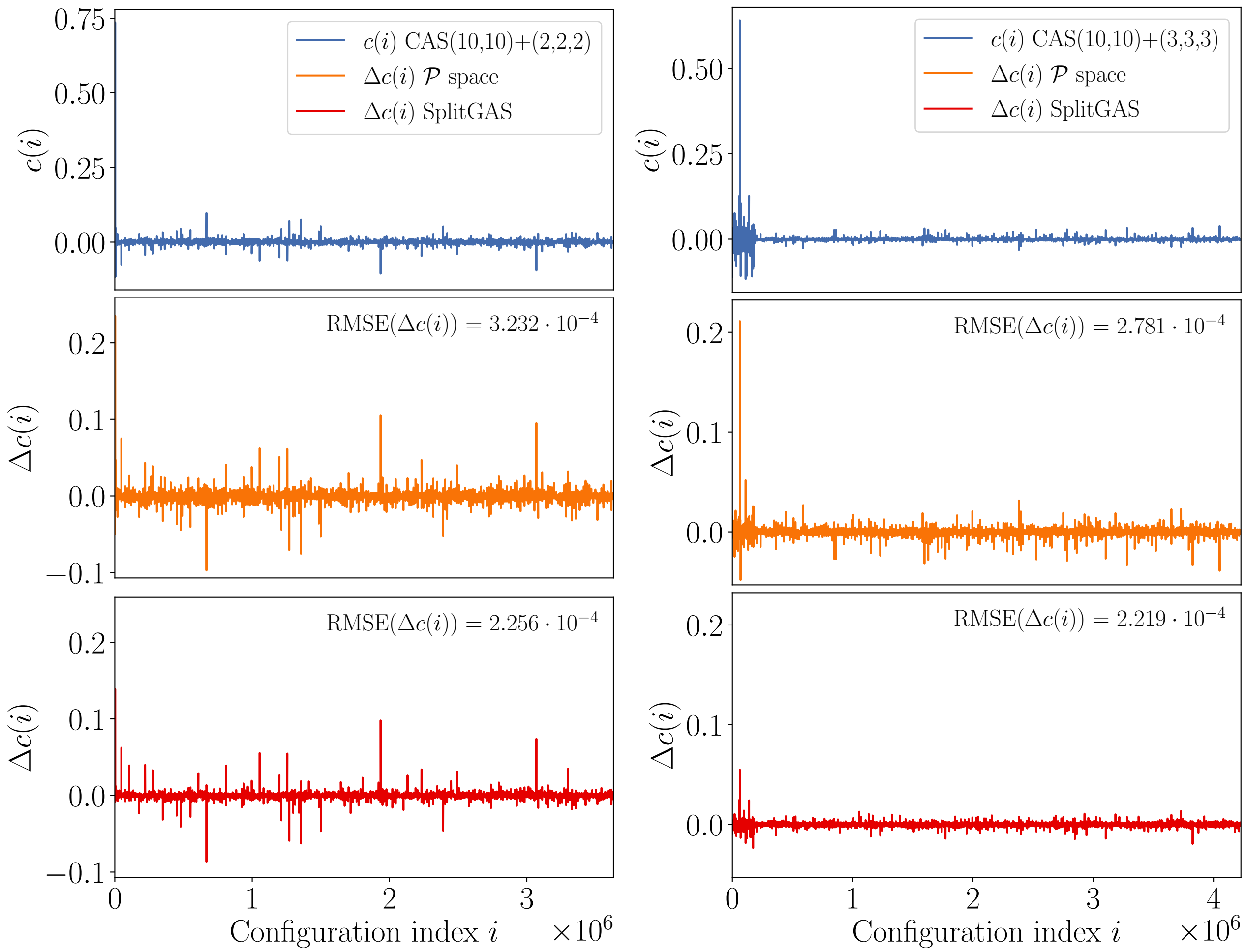

(a) $\mathcal{T}$ =CAS(10,10)+(2,2,2)

(b) $\mathcal{T}$ = CAS(10,10)+(3,3,3)

Figure S2: Wave function coefficients of two total spaces and the deviation of the respective $\mathcal{P}$ and SplitGAS wave functions from the total wave function.

An analysis of the full time averaged wave function was omitted due to scaling issues as it involved around 50 million configurations.
